# Identification of patient-specific CD4^+^ and CD8^+^ T cell neoantigens through HLA-unbiased genetic screens

**DOI:** 10.1038/s41587-022-01547-0

**Published:** 2023-01-02

**Authors:** Chiara M. Cattaneo, Thomas Battaglia, Jos Urbanus, Ziva Moravec, Rhianne Voogd, Rosa de Groot, Koen J. Hartemink, John B. A. G. Haanen, Emile E. Voest, Ton N. Schumacher, Wouter Scheper

**Affiliations:** 1 Department of Molecular Oncology and Immunology, The Netherlands Cancer Institute, Amsterdam, The Netherlands; 2 Oncode Institute, Utrecht, The Netherlands; 3 Department of Genomics of Cancer and Targeted Therapies, IFOM, FIRC Institute of Molecular Oncology, Milan, Italy; 4 Department of Hematopoiesis, Sanquin Research, Amsterdam, The Netherlands; 5 Department of Hematology, Leiden University Medical Centre, Leiden, The Netherlands; 6 Department of Surgery, The Netherlands Cancer Institute, Amsterdam, The Netherlands; 7 Department of Medical Oncology, The Netherlands Cancer Institute, Amsterdam, The Netherlands

**Keywords:** Tumour immunology, Immunotherapy, Functional genomics

## Abstract

Cancer neoantigens that arise from tumor mutations are drivers of tumor-specific T cell responses, but identification of T cell-recognized neoantigens in individual patients is challenging. Previous methods have restricted antigen discovery to selected HLA alleles, thereby limiting the breadth of neoantigen repertoires that can be uncovered. Here, we develop a genetic neoantigen screening system that allows sensitive identification of CD4^+^ and CD8^+^ T cell-recognized neoantigens across patients’ complete HLA genotypes.

## Abstract (web summary)

Patient-specific tumor neoantigens are recognized by T cells.

## Main

Cancer immunotherapies that aim to harness the antitumor activity of T cells have shown impressive clinical results in a subset of patients with cancer, and accumulating evidence suggests that the efficacy of these therapies is driven largely by T cells that recognize cancer neoantigens that result from patient-specific nonsynonymous tumor mutations^1^. Consequently, there is a strong interest in developing approaches to specifically boost the number or activity of neoantigen-reactive T cells in individual patients. However, identification of T cell-recognized neoantigens is challenging due to their patient-specific nature^2^. Previous antigen discovery methods have been limited by relying on the use of single or selected HLA alleles^3–7^ and are therefore not straightforwardly compatible with identifying T cell (neo)antigens across the complete HLA haplotypes of individual patients with cancer. Moreover, while CD4^+^ T cells have important roles in tumor control and response to immunotherapy^8–11^, previous methods have focused primarily on the identification of CD8^+^ T cell-recognized neoantigens. Thus, experimental tools are required to enable the routine and HLA-unbiased identification of CD4^+^ and CD8^+^ T cell-recognized neoantigens in individual patients.

**Fig. 1:**
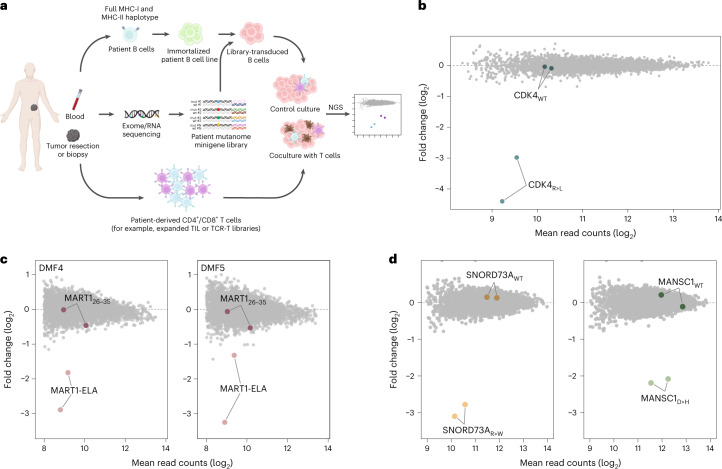
Overview and validation of neoantigen discovery technology. **a**, Schematic overview of the methodology. **b**, Antigen discovery screen of CD8^+^ TCR #53 T cells against immortalized HLA-A*02:01^+^ B cells transduced with a model antigen library of *n* = 4,764 minigenes. Dots represent individual minigenes. Fold change, defined as the relative abundance of minigenes in the presence of TCR #53 T cells compared with mock T cells, and mean normalized read counts are plotted for each individual minigene. Minigenes encoding the model CDK4 mutant and WT epitopes are highlighted. CDK4_R24L_ minigenes: *P* = 9.4 × 10^−43^, *P* = 1.5 × 10^−9^. *P* values were generated using the DESeq2 Wald test (one-sided) and adjusted for several comparisons. **c**, Antigen screen using CD8^+^ T cells expressing either the DMF4 (left panel) or DMF5 (right panel) TCR against model library-expressing HLA-A*02:01^+^ B cells. Data are plotted as in **b** with fold change showing relative minigene abundance when exposed to either DMF4 or DMF5 TCR T cells as compared with mock T cells. DMF4 MART1-ELA minigenes: *P* = 1.9 × 10^−12^, *P* = 1.7 × 10^−9^; DMF5 MART1-ELA minigenes: *P* = 4.1 × 10^−14^, *P* = 8.8 × 10^−7^. *P* values were generated as in **b**. **d**, Antigen screen using CD4^+^ T cells expressing patient-derived TCRs specific for the MHC class II-restricted SNORD73A_R>W_ and MANSC1_D>H_ against patient-matched immortalized B cells transduced with the CD74 signal-fused model library. Data are plotted as in **b** with fold change showing relative minigene abundance in the presence of CD4^+^ SNORD73A TCR or MANSC1 TCR T cells relative to mock T cells. SNORD73A_R>W_: *P* = 2.1 × 10^−53^; *P* = 2.5 × 10^−44^; MANSC1_D>H_: *P* = 2.1 × 10^−26^, *P* = 3.2 × 10^−8^. *P* values were generated as in **b**.

Here, we present a high-throughput genetic system for the personalized identification of CD4^+^ and CD8^+^ T cell-recognized (neo)antigens (Fig. 1a). In this method, termed *HANSolo* (HLA-Agnostic Neoantigen Screening), patient-matched, Bcl-6/xL-immortalized B cell lines are engineered to express large libraries of minigenes that encode candidate T cell antigens. As the resulting B cells are fully MHC class I and class II proficient, this enables the unbiased screening of T cell specificities across the complete MHC class I and class II genotypes of individual patients using T cell pools as selective pressure. To this purpose, antigen library-expressing B cells are coincubated with patient T cell populations of interest (for example, tumor-infiltrating lymphocytes (TIL) or T cells engineered to express patient-derived T cell receptors (TCRs)^12^), and antigen hits are identified by next-generation sequencing to measure the depletion of those B cells that express T cell-recognized epitopes.

To first evaluate the feasibility and sensitivity of our method, we took advantage of well-described HLA-A*02:01-restricted TCRs specific for either the CDK4_R24L_ neoantigen (TCR #53)^13^ or for the melanocyte differentiation antigen-derived MART1_26-35_ epitope (TCRs DMF4 and DMF5)^14^ (Supplementary Fig. 1). Activity of the CDK4_R24L_ neoantigen-specific TCR should result in strong depletion of B cells expressing the mutant, but not the wild-type (WT), CDK4 sequence. TCR DMF4 has an affinity towards the MART1 self antigen that is around fivefold lower as compared with the DMF5 TCR^14,15^, providing a means to assess the sensitivity of the method in the context of weak T cell–target cell interactions. Furthermore, the use of the parental MART1 epitope as well as a previously identified variant with increased affinity for MHC-I^16^ (here referred to as MART1-ELA) should allow one to determine whether the level of epitope presentation can be gauged from screening data. To provide first proof-of-concept, we designed a model antigen library with a complexity (4,764 minigenes) that would be sufficient to enable the screening of the entire mutational repertoire of human tumors with the highest mutational burden, such as melanomas, lung tumors and microsatellite-instable tumors^17^. Individual MHC class I-restricted antigens, including the CDK4_R24L_ and MART1 antigens and immunodominant epitopes of EBV, CMV and influenza, as well as MHC class II-restricted neoantigens (Supplementary Table 1) were expressed as minigenes, each coupled to two unique barcode identifiers to provide internal replicate measurements. Subsequently, HLA-A*02:01-positive immortalized B cells were created and modified to express the epitope library.

Following optimization of conditions to ensure maximal sensitivity of antigen screens (Supplementary Fig. 2), screening of this proof-of-concept library with T cells expressing the CDK4_R24L_-specific TCR resulted in clear depletion of CDK4_R24L_-expressing B cells, but crucially not B cells expressing the WT CDK4 minigene (Fig. 1b). Furthermore, B cells expressing the MART1-ELA epitope showed substantial depletion after exposure to T cells transduced with the MART1-specific DMF4 or DMF5 TCRs (Fig. 1c). Notably, the level of depletion mediated by the low affinity DMF4 TCR was comparable with that of the DMF5 TCR. Moreover, when using the high affinity 1D3 TCR^18^, depletion was observed for both MART1 epitopes but was substantially stronger for the MART1-ELA epitope (Supplementary Figs. 1 and 3). Next, to test whether this system allows the profiling of the antigen-specificities of T cell populations in which T cells specific for a given antigen make up only a minority of the total T cell pool (such as patient TIL cultures, or donor T cells expressing libraries of patient-derived TCRs), we mixed T cells expressing either the DMF4, DMF5 or 1D3 TCR with mock-transduced T cells, such that MART1-specific T cells represented 10%, 1%, 0.3% or 0.1% of total T cells. Analysis of epitope abundance after exposure to these different T cell populations demonstrated that the MART1-ELA epitope was robustly identified when cognate TCR-expressing T cells comprised as little as 0.1–0.3% of all T cells (Supplementary Fig. 3). Depletion of the native MART1 epitope was detected only when using the high affinity 1D3 TCR. Together, these data demonstrate that our genetic screening methodology allows the efficient discovery of MHC class I-restricted T cell (neo)antigens from large antigen libraries. Furthermore, the technology allows one to distinguish high and low avidity TCR-pMHC interactions and genetic screens may be performed with clonally diverse T cell populations.

A substantial fraction of T cell-recognized cancer neoantigens is restricted by MHC class II molecules, and CD4^+^ T cells recognizing such MHC class II-restricted neoantigens contribute to tumor control^8–11^. To test the suitability of HANSolo for the discovery of MHC class II-restricted neoantigens, we explored a previously established engineering method that routes individual minigene products through both the MHC class I and class II presentation pathways. In line with expectations, fusion of neoantigen-encoding minigenes to the sorting signal of the invariant chain (CD74) resulted in robust activation of both CD4^+^ and CD8^+^ neoantigen-specific T cells (Supplementary Fig. 4), and this universal antigen expression system was therefore selected for further use. We next took advantage of two MHC class II-restricted neoantigen-specific TCRs that were isolated from tumor-infiltrating T cells of a melanoma patient (Supplementary Fig. 4), transduced both TCRs into donor CD4^+^ T cells and expressed the model antigen library in patient-matched immortalized B cells. Screening of library-expressing B cells with T cells expressing either MHC class II-restricted TCR resulted in the notable depletion of B cells that expressed the cognate neoantigen, but not its WT counterpart (Fig. 1d). Furthermore, the use of CD4^+^ T cell populations in which T cells expressing either of the MHC-II-restricted neoantigen-specific TCRs were present at low frequency demonstrated clear depletion of the relevant neoantigens at antigen-specific CD4^+^ T cell frequencies as low as 0.3–1% (Supplementary Fig. 5).

**Fig. 2:**
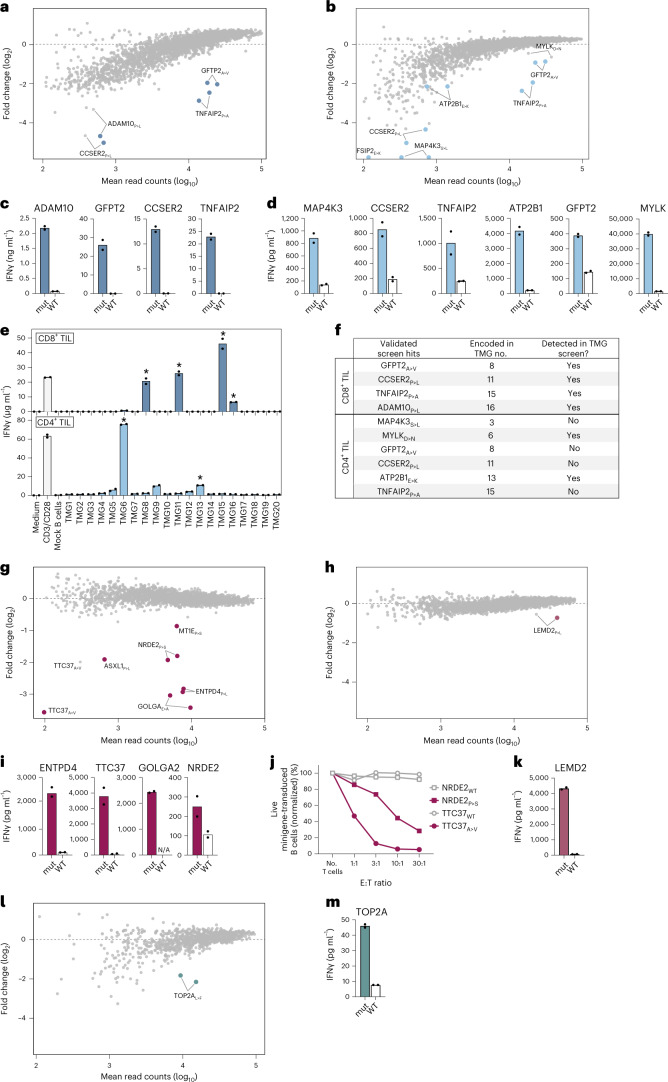
Personalized and HLA-agnostic neoantigen screening of patient-derived CD4^+^ and CD8^+^ T cells. **a**,**b**, Nonsynonymous tumor mutations of patient NKIRTIL063 were identified by exome and RNA sequencing and used to design a personalized mutanome minigene library consisting of *n* = 2,762 unique minigenes. Patient B cells were immortalized, transduced with the mutanome library and screened with in vitro-expanded tumor-infiltrating CD8^+^ (**a**) and CD4^+^ (**b**) T cells. Fold change represents relative minigene abundance in cultures with or without patient T cells. Screen hits were defined as outlined in Methods and are marked by colored dots. **c**,**d**, Validation of neoantigen hits identified in **a** and **b** by incubating patient CD8^+^ (**c**) or CD4^+^ (**d**) with autologous B cells expressing either neoantigen hits (mut) or respective WT sequences as single minigenes. T cell activation was assessed by measuring IFNγ levels in supernatants. Dots represent technical replicates. **e**, NKIRTIL063 CD8^+^ and CD4^+^ T cells were incubated with patient B cells expressing indicated TMG constructs, followed by measuring IFNγ concentrations in culture supernatants. Asterisks indicate TMG constructs that encode a neoantigen identified using the antigen screens in **a**–**d**. Dots represent technical replicates. **f**, Summary of NKIRTIL063 neoantigens identified using the HANSolo screens and TMG approach. **g**,**h**, Patient NKIRTIL027 immortalized B cells were transduced with the patient mutanome library (*n* = 2,586 minigenes) and screened using in vitro-expanded NKIRTIL027 CD8^+^ (**g**) and CD4^+^ (**h**) tumor-infiltrating T cells. Fold change depicts relative minigene abundance in cultures with or without patient T cells. Screen hits are marked by colored dots. **i**,**j**, NKIRTIL027 CD8^+^ TIL screen hits were validated by incubating patient T cells with matched B cells expressing the single mutant or corresponding WT sequences, and measuring IFNγ levels in supernatants (**i**) or killing of transduced B cells after exposure to patient T cells at indicated effector:target (E:T) ratios (**j**). Dots represent technical replicates. N/A, not available. **k**, The NKIRTIL027 CD4^+^ T cell screen hit was validated as in **i**. **l**,**m**, Neoantigen specificities of patient ITO34 CD8^+^ TIL were screened against the patient mutatome library (*n* = 952 minigenes) and validated as in **g** and **i**.

As compared with previously developed genetic screening technologies, HANSolo has the advantage of allowing the identification of T cell epitopes restricted by any of the class I or II alleles of an individual patient. To demonstrate the utility of such unbiased screening, we first focused on analysis of neoantigen reactivity among intratumoral T cells in a patient with metastatic melanoma (patient NKIRTIL063). CD4^+^ and CD8^+^ T cell cultures were generated by in vitro expansion of TIL, and both resulting T cell populations possessed cytotoxic potential, as measured by degranulation potential upon polyclonal stimulation (Supplementary Fig. 6). In parallel, nonsynonymous mutations in protein-coding genes were identified by exome and RNA sequencing, yielding 685 nonsynonymous expressed tumor variants, and a library of 2,762 minigenes that encoded all identified tumor mutations, as well as their corresponding WT sequences, was generated and expressed in autologous immortalized B cells. Screening of this patient mutanome library with in vitro-expanded CD8^+^ TIL revealed TIL reactivity towards four neoantigens (Fig. 2a). Importantly, no reactivity against the corresponding WT minigenes in the library was detected. Furthermore, screening the same neoantigen library with CD4^+^ TIL yielded reactivity against six neoantigens (Fig. 2b). Both minigenes encoding the tumor variant MYLK_D>N_ showed reproducible low-level depletion after coculture with CD4^+^ TIL, and this variant was therefore considered a putative screen hit. Recognition of screen-identified neoantigens, but not WT counterpart sequences, was subsequently validated upon expression of the individual sequences in patient B cells, resulting in confirmed CD4^+^ and CD8^+^ TIL reactivity towards 10 out of 11 identified screen hits (Fig. 2c,d and Supplementary Fig. 6). Notably, three neoantigens—GFPT2_A>V_, TNFAIP2_P>A_ and CCSER2_P>L_—were recognized by both CD8^+^ and CD4^+^ TIL of this patient.

To assess the sensitivity of our method in comparison with other available neoantigen discovery methods, we next analyzed neoantigen reactivity among CD4^+^ and CD8^+^ TIL of patient NKIRTIL063 using the previously established tandem minigene (TMG) approach^4,19^, in which generally ten minigenes are concatenated and expressed as a single transgene in separate pools of antigen-presenting cells. To screen neoantigen specificities of patient NKIRTIL063 CD4^+^ and CD8^+^ TIL using TMGs within a reasonable timeframe, 200 of the 685 mutations were selected on the basis of expression level and mutation clonality and used to generate 20 pools of patient B cells. Incubation of these cell pools with either CD4^+^ or CD8^+^ TIL of patient NKIRTIL063 revealed notable reactivity of CD4^+^ TIL to three TMGs (#6, #9 and #13) and reactivity of CD8^+^ TIL to four TMGs (#8, #11, #15 and #16) (Fig. 2e). Recognition of six out of seven TMGs was mediated by neoantigens identified before using the genetic library screens, as demonstrated by a subsequent deconvolution step (Supplementary Fig. 6). TMG#9 did not encode a neoantigen hit from our screens but did elicit low-level reactivity of CD4^+^ TIL. Conversely, the TMG screen failed to identify four CD4^+^ TIL-recognized neoantigens that were detected using the HANSolo screens (Fig. 2f), demonstrating the potential of the method to mine patient neoantigens with increased depth compared with existing methodologies.

Next, to assess the value of the developed system for the routine discovery of neoantigens across patients with cancer, we mapped neoantigen specificities in three additional patient samples. Tumor mutations were identified in an additional melanoma tumor (NKIRTIL027; 660 nonsynonymous expressed mutations) and used to construct a patient mutanome library of 2,562 minigenes. Screening the neoantigen specificities of CD4^+^ and CD8^+^ TIL resulted in six putative CD8^+^ TIL-recognized neoantigens (Fig. 2g) and one neoantigen recognized by CD4^+^ TIL (Fig. 2h), and recognition of these epitopes was confirmed for five out of seven neoantigens (Fig. 2i–k and Supplementary Fig. 7). In addition, as observed in genetic screens using model antigens and TCRs, the level of epitope depletion in this patient screen correlated with the capacity of patient T cells to produce interferon gamma (IFNγ) in response to minigene-expressing B cells and kill such cells (Fig. 2i,j). We next analyzed neoantigen specificities of intratumoral CD4^+^ and CD8^+^ T cells in a nonsmall cell lung tumor (patient ITO34; 231 mutations), resulting in the detection of CD8^+^ TIL reactivity against one neoantigen (Fig. 2l,m and Supplementary Fig. 8). Recently, strategies that enrich T cell populations for tumor-specific T cells by culture with patient tumor organoids^20,21^ or antigen-expressing APCs^22^ have been reported. To assess whether such strategies may complement our methodology, for instance, in settings where fresh tumor material for the generation of TIL cultures is unavailable, we applied our screening method to a microsatellite-instable colorectal tumor (ITO66; 1,834 mutations). For this purpose, the patient mutanome was screened using a CD8^+^ T cell product that was generated by ex vivo culture of patient peripheral blood mononuclear cells (PBMCs) with matched tumor organoids, resulting in the identification of two CD8^+^ T cell-recognized neoantigens (Supplementary Fig. 9). Thus, the use of our screening methodology enabled the successful identification of patient neoantigens in all four tested patients.

Collectively, these data demonstrate the feasibility of personalized and HLA-agnostic discovery of CD4^+^ and CD8^+^ T cell neoantigens from large genetic libraries. Benchmarking against the existing TMG method demonstrated enhanced sensitivity of our approach, in particular for the discovery of CD4^+^ T cell-recognized neoantigens, while enabling substantially improved throughput. From a translational perspective, identified neoantigens may be used to select TCRs for use in next-generation TCR gene therapies or may be utilized in patient-specific cancer vaccines^22–26^. Of note, state-of-the-art algorithms that predict the immunogenicity of tumor mutations for use in personalized neoantigen vaccines ranked only 3 out of all 14 identified patient neoantigens as actionable vaccination targets (Supplementary Table 2), underlining the value of approaches that allow the unbiased and functional identification of patient neoantigens. With the current next-generation sequencing and DNA synthesis technologies and dedicated screening workflows, our system enables patient neoantigen discovery within 10 weeks (Supplementary Fig. 10), a timespan that is compatible with the production of personalized immunotherapies^24^.

## Methods

### Antibodies

The following antibodies were used for flow cytometry: CD3-PerCP-Cy5.5 (clone SK7; eBioscience; used 1:20); CD4-FITC (clone RPA-T4; BD Biosciences; used 1:20), CD4-APC (clone RPA-T4; BD Biosciences; used 1:30), CD4-BV421 (clone SK3, Biolegend; used 1:100), CD8-BV421 (clone RPA-T8; BD Biosciences; used 1:50), CD14-APC-H7 (clone MoP9, BD Biosciences; used 1:100), CD16-APC-H7 (clone 3G8, BD Biosciences; used 1:100), CD19-FITC (clone 4G7, BD Biosciences; used 1:30), CD137-BV421 (clone 4B4-1; Biolegend; used 1:200), CD137-APC (clone 4B4-1; BD Biosciences; used 1:30), OX40-PE-Cy7 (clone Ber-ACT35, Biolegend), CD107-PE (clone H4A3, BD Biosciences; used 1:150) and PE-conjugated anti-mouse TCRβ constant domain (clone H57-597; BD Biosciences; used 1:150). The viability stain IR-Dye (Thermo Fisher, used 1:2,000) was used to identify live cells.

### Generation of patient T cell products, Bcl-6/Bcl-xL-immortalized B cells and tumor organoids

Tumor tissue and PBMCs were collected from patients treated at the Netherlands Cancer Institute—Antoni van Leeuwenhoek Hospital (NKI-AVL) with written informed consent and in accordance with guidelines of the Medical Ethical Committee. The study protocol was approved by the Medical Ethical Committee of the NKI-AVL. Fresh tumor tissue obtained by surgical resection was mechanically disrupted and digested overnight in RPMI 1640 medium (Life Technologies) supplemented with 1 mg ml^−1^ collagenase type IV (BD Biosciences), penicillin-streptomycin (Roche) and 0.01 mg ml^−1^ pulmozyme (Roche).

For patients NKIRTIL027, NKIRTIL063 and ITO34, TIL cultures were generated by culturing tumor digest suspensions in T cell medium (RPMI 1640 medium supplemented with 10% human AB serum (Life Technologies), penicillin-streptomycin, l-glutamine (Life Technologies)), supplemented with 6,000 U ml^−1^ IL-2 (Proleukin, Novartis) for 2–4 weeks. Obtained TIL cultures were subsequently stained with IR-Dye and antibodies against CD3, CD4 and CD8, and single CD3^+^CD4^+^ and CD3^+^CD8^+^ T cells were sorted using a FACSAria Fusion cell sorter (BD Biosciences). Isolated CD4^+^ and CD8^+^ T cells were expanded using the rapid expansion protocol (REP), using 30 ng ml^−1^ anti-CD3 antibody (clone OKT-3; eBioscience) and 3,000 U ml^−1^ IL-2 in a 1:1 mixture of RPMI 1640 and AIM-V medium (Gibco) supplemented with 5% human AB serum, in the presence of irradiated (40 Gy) allogeneic PBMCs (200:1 feeder/T cell ratio). After 7 days of REP culture, medium was refreshed with medium and IL-2 every 2 days. Purity of the resultant CD4^+^ and CD8^+^ T cell populations was confirmed by flow cytometry at day 14 after start of REP (routinely >99%), and cells were subsequently either used directly in antigen discovery screens or cryopreserved in liquid nitrogen. Data from flow cytometry experiments was acquired using FACSDiva software and analyzed using Flowjo (BD Biosciences).

Immortalized patient B cell lines were generated by retroviral transduction with Bcl-6/Bcl-xL^27^. Patient PBMCs were isolated from peripheral blood by Ficoll-Paque density gradient separation and stained with IR-Dye and antibodies against CD3, CD14, CD16 and CD19. Single IR-Dye^−^CD3^−^CD14^−^CD16^−^CD19^+^ cells were sorted using a FACSAria Fusion cell sorter and stimulated for 36 h with irradiated (55 Gy) CD40L^+^ mouse L cells in B cell medium (IMDM medium (Gibco) supplemented with penicillin-streptomycin, 10% heat-inactivated fetal bovine serum (Sigma-Aldrich) and 50 ng ml^−1^ IL-21 (Peprotech)), followed by retroviral transduction of Bcl-6 and Bcl-xL. The Bcl-6/Bcl-xL-encoding vector also encodes GFP to allow evaluation of transduction efficiency. Bcl-6/Bcl-xL-immortalized (GFP^+^) B cells were cultured in B cell medium and were stimulated every week by addition of irradiated CD40L^+^ L cells. Medium and IL-21 were refreshed every 3–4 days.

For patient ITO66, tumor organoids were established^20,21^. Tumor-reactive patient T cells were generated by coculturing PBMCs and tumor organoids as follows. Following incubation with 200 ng ml^−1^ IFNγ (Peprotech) for 24 h, tumor organoids were dissociated into single-cell suspensions using TripLE Express (Gibco). Tumor organoid cells were mixed with patient PBMCs (20:1 PBMC/tumor cell ratio) and 1 × 10^5^ PBMC were seeded in each well of a U-bottom 96-well plate precoated with 5 μg ml^−1^ anti-CD28 antibody (clone CD28.2; eBioscience). Coculture medium consisted of T cell medium supplemented with 150 U ml^−1^ IL-2 and 20 µg ml^−1^ anti-PD1 blocking antibody (clone 5C4; kindly provided by Merus). Coculture medium was refreshed every 2–3 days. PBMCs were harvested and restimulated every 7 days by replating with fresh tumor organoid cells.

### Retroviral transduction of TCRs

Codon-optimized TCR α and β variable sequences (encompassing V-CDR3-J domains) of selected TCRs were gene-synthesized (Twist Biosciences) and subcloned into a modified pMP71 retroviral vector^12^. This vector contains mouse TCR constant regions to reduce mispairing of introduced and endogenous TCR chains, as well as the puromycin N-acetyltransferase resistance gene. Retrovirus was produced by transfecting FLY-RD18 packaging cells with pMP71-TCR plasmid DNA using Xtremegene 9 transfection reagent (Roche). In parallel, healthy donor PBMCs (Sanquin Blood Bank) were separated into CD8^+^ and CD8^−^ (for transduction with MHC class I- and MHC class II-restricted TCRs, respectively) cells using the CD8^+^ T Cell Isolation Kit (Miltenyi Biotec). Isolated cell fractions were stimulated with CD3/CD28 Dynabeads (Life Technologies) in T cell medium with 150 U ml^−1^ IL-2. After 48 h, retroviral supernatants were collected and used to infect prestimulated CD8^−^/CD8^+^ PBMCs by spinoculation (2,000 *g* for 90 min) in Retronectin (Takara)-coated plates. Transduction efficiency was measured 72 h later by staining with an anti-mouse TCRβ constant domain antibody and analysis by flow cytometry. TCR-transduced T cells were then selected with 2.5 µg ml^−1^ puromycin (Gibco) for 48 h and received fresh medium and IL-2 every 3–4 days. After 12–14 days of culture, transduced T cells were expanded using the REP as described above.

### T cell activation assays

Reactivity of TCR-transduced donor T cells was determined by coincubating T cells and target cells for 18–24 h in U-bottom 96-well plates (1:1 T cell/target cell ratio) in T cell medium. Incubation of T cells without target cells, and in the presence of 50 ng ml^−1^ phorbol 12-myristate 13-acetate (Sigma-Aldrich) and 1 µg ml^−1^ ionomycin (Sigma-Aldrich) served as negative and positive controls, respectively. Following incubation, cells were stained with IR-Dye and antibodies against CD3, CD4, CD8 and the activation markers CD137 or OX40 and analyzed by flow cytometry. When T cell reactivity towards tumor organoids was tested, IFNγ-pretreated organoids were incubated with T cells in the presence of 20 µg ml^−1^ anti-PD1 blocking antibody (Merus) in anti-CD28 antibody precoated plates.

The cytotoxic capacity of T cells was assessed by coincubating T cells and target cells for 72 h in 96-well plates at a T cell/target cell ratio of 5:1, unless indicated otherwise. Target cells cultured in the absence of T cells served as negative control. Following incubation, 7.46 µm AccuCount blank counting beads (Spherotech) were added to individual cultures to enable quantification of remaining live target cells. Cells were subsequently harvested, stained with 4,6-diamidino-2-phenylindole and anti-CD3 antibody, and measured by flow cytometry. When cytotoxicity against tumor organoids was assessed, IFNγ-pretreated organoids were incubated with T cells in the presence of 20 µg ml^−1^ anti-PD1 blocking antibody (Merus) and 10 µM Y-27632 in anti-CD28 antibody precoated 96-well plates. Where indicated, target cells were incubated with 50 µg ml^−1^ MHC class I blocking antibody (clone W6/32) for 30 min at 37 °C before incubation with T cells. Data from functional T cell assay was analyzed using Graphpad Prism v.9.

### Exome and RNA sequencing

Tumor genomic DNA and RNA was extracted from formalin-fixed paraffin embedded tumor material using the AllPrep DNA/RNA kit (Qiagen). For patient ITO66, genomic DNA and RNA were isolated from tumor organoids. Genomic DNA of patient PBMCs was extracted using the DNeasy Blood & Tissue kit (Qiagen). Exome enrichment was performed using the SureSelect XT2 Human All Exon V6 kit (Agilent) and strand-specific libraries were generated using the TruSeq Stranded mRNA sample preparation kit (Illumina) according to the manufacturer’s instructions. Resulting libraries were sequenced on HiSeq 2500 or NovaSeq 6000 DNA analyzers (Illumina). Whole-exome and RNA sequencing was processed using bcbio-nextgen. Briefly, DNA reads were mapped against GRCh38 using Burrows–Wheeler aligner (BWA), duplicates were marked with Picard MarkDuplicates and low complexity regions were excluded. Somatic and germline mutations were identified using Mutect2 and HaplotypeCaller, respectively, followed by annotation by SnpSift. RNA reads were quality filtered and mapped with STAR or TopHat2, transcript-level expression was quantified by Salmon and gene fusions were determined by Arriba^12,21^.

### Antigen library design

To design the model antigen library used to validate the screening system, protein sequences of genes encoding known human nonmutated cancer regression antigens, as well as selected immunodominant epitope-encoding genes of Epstein-Barr virus, cytomegalovirus and influenza, were collected from the Uniprot database (https://www.uniprot.org/) (Supplementary Table 1). Protein sequences were reverse-translated and codon-optimized, and resulting nucleotide sequences were segmented into 93 nucleotide (nt) minigenes with 45 nt overlap between neighboring minigenes. In addition, a set of previously characterized neoantigens was included, all encoded by 93 nt minigenes in which the mutant codon was flanked on either side by 45 nt of the relevant nonmutant gene sequence. Minigene sequences encoding the corresponding nonmutated peptides were included for each model neoantigen. A stop codon was added directly following each minigene sequence, and internal *Bbs*I recognition sites were removed without altering the encoded peptide sequences. Each 93 nt sequence was duplicated for a total of 4,764 sequences, and a unique 12 nt barcode sequence was incorporated into each minigene sequence following the stop codon. The resulting sequences were flanked by sequences to enable PCR amplification and subcloning using *Bbs*I (New England Biolabs) into a pMSCV retroviral vector that also encodes the puromycin N-acetyltransferase resistance gene and mCherry (pMSCV-puroR-mCherry).

To design NKIRTIL027 and NKIRTIL063 patient mutanome libraries, all single nucleotide variants (SNVs) and frameshifting indels with confirmed RNA expression within tumor cells were encoded as 93 nt minigenes. RNA sequencing data of tumor ITO34 was unavailable, and the library was designed without taking RNA expression of tumor variants into account. For SNVs, minigenes were designed that encoded peptides in which the mutant codon was flanked on either side by 45 nt of the relevant nonmutant gene sequence. In the case of frameshifting indels, or when SNVs resulted in loss of a stop codon, the newly formed open reading frame was segmented in 93 nt minigenes with 45 nt overlap between adjacent minigenes. Minigenes encoding corresponding WT sequences were included for all tumor variant minigenes. Minigenes encoding the MART1_26–35_ and CDK4_R24L_ epitopes were included in all libraries as internal controls. Internal *Bbs*I recognition sites were removed without altering encoded peptide sequences, and minigenes were flanked by sequences for PCR amplification and subcloning as described above. For patient ITO66, the mutanome library was designed to encode tumor variants as 63 nt minigenes, and no corresponding WT minigenes were included. All minigene libraries were synthesized by Twist Biosciences.

### Generation of a universal antigen expression vector

To establish a library expression system that enables the concurrent processing and presentation of minigene products through both the MHC class I and class II pathways, constructs were designed in which a TMG encoding two previously identified neoantigens recognized by either CD4^+^ or CD8^+^ TIL of patient NKIRTIL027 (LEMD2_P>L_ (ref. ^28^) and TTC37_A>V_ (unpublished data), respectively) was either fused or not fused to the signal sequence of CD74 (Supplementary Fig. 4)^29^. Codon-optimized constructs were synthesized (Twist Biosciences) and subcloned into the retroviral pMSCV-puroR-mCherry vector. NKIRTIL027 immortalized B cells were transduced with TMG constructs, selected to over 90% purity (by measuring mCherry expression) with 5 μg ml^−1^ puromycin and incubated with NKIRTIL027 CD4^+^ or CD8^+^ TIL at a ratio of 1:1 for 48 h in T cell medium with 30 U ml^−1^ IL-2. T cell activation was subsequently assessed by measuring IFNγ levels in the culture supernatant using the Cytometric Bead Array kit (BD Biosciences), following the manufacturer’s instructions.

### Library cloning and transduction

Oligonucleotide libraries were amplified by 12 cycles of PCR using Phusion High-Fidelity DNA Polymerase (New England Biolabs) and primers Preamp Forward (5′-ACTGTCAGAAGACTGCAAGC-3′) and Preamp Reverse (5′-TGACAGCGAAGACCATAGTG-3′). For first proof-of-concept screening experiments using MHC class I-restricted TCRs, the amplified model antigen library was cloned by Golden Gate assembly using *Bbs*I into the pMSCV-puroR-mCherry retroviral vector. For all other screens, amplified libraries were cloned into the pMSCV-puroR-mCherry vector modified to include the sorting sequence of CD74. Subcloned libraries were amplified using Endura electrocompetent cells (Lucigen) and library DNA was extracted using the PureLink HiPure Maxiprep kit (Invitrogen). During all cloning steps, a library representation of at least 100× was maintained.

Libraries were retrovirally transduced in duplicate into immortalized B cell lines, as described above. To ensure single retroviral integrations, B cells were transduced at an infection rate of less than 10%. One day after transduction, B cells were transferred to B cell medium in the presence of irradiated CD40L^+^ L cells. Transduction efficiency was assessed 3 days post-transduction by measuring mCherry expression by flow cytometry, followed by selection with 5 µg ml^−1^ puromycin for 2 days and expansion of the B cell cultures until used in screens.

### Antigen discovery screens

For proof-of-concept screens using MHC class I-restricted TCRs, the antigen library encoding known cancer regression antigens was transduced into a previously immortalized HLA-A*02:01^+^ patient B cell line (OVC21)^12^. Library-expressing B cells were coincubated in duplicate with donor CD8^+^ T cells transduced with the CDK4_R24L_-specific TCR #53 or MART_26–35_-specific TCRs DMF4, DMF5 or 1D3 (all HLA-A*02:01-restricted) in T cell medium with 25 U ml^−1^ IL-2 at a T cell:B cell ratio of 5:1 and at a density of 2 × 10^6^ total cells cm^−2^. Cultures were resuspended on day 1 and 2 of the experiment. For screens using patient-derived MHC class II-restricted neoantigen-specific TCRs, the model library was transduced into patient-matched immortalized B cells (patient NKIRTIL017), and library-expressing B cells were cocultured with donor CD4^+^ T cells transduced with either the MANSC1_D>H_- or SNORD73A_R>W_-specific TCR as described above. To simulate screening conditions using clonally diverse T cell populations, TCR-expressing T cells were mixed with donor-matched mock-transduced T cells at indicated ratios. Library coverage of at least 300× was maintained in all experiments. After 72 h of coincubation, cells were washed in PBS, and cell debris was removed by either Ficoll-Paque density gradient separation or using the Dead Cell Removal kit (Miltenyi Biotec). Isolated cells were subsequently resuspended in DirectPCR Lysis Reagent (Viagen) containing 500 µg ml^−1^ proteinase K and lysed by incubation at 55 °C for 60 min, 85 °C for 30 min and 94 °C for 5 min. Minigene sequences were then amplified by PCR using NEBNext Ultra II Q5 Master Mix (New England Biolabs), using the following primers:

Prep-I Forward (for screens with MHC class I-restricted TCRs):

5′-CAAGCAGAAGACGGCATACGATGGAGGAGAACCCTGGACCTACAAGC-3′

Prep-II Forward (for all other screens):

5′-CAAGCAGAAGACGGCATACGACCTGCGGATGAAGCTGCCCG-3′

Prep Reverse:

5′-AATGATACGGCGACCACCGAGATCTACACTCTTTCCCTACACGACGCTCTTCCGATCTNNNNNNNG ATCCGACTCGGTGCCACTTTTTCAAC-3′

The 7-nt stretch of N nucleotides indicates a unique barcode sequence used to enable the multiplexed preparation of sequencing libraries. Following PCR, samples were pooled equimolarly and run on a 1% agarose gel to separate minigene amplicons from potential primer dimers. Minigene amplicons were extracted from gel using the Monarch DNA Gel Extraction Kit (New England Biolabs) and deep sequenced on an Illumina HiSeq 2500 Sequencing system (single read 65 bp). Sequencing data were deposited in the National Center for Biotechnology Information (NCBI) Sequence Read Archive under accession code PRJNA884260 (ref. ^30^).

For patient neoantigen screens, mutanome libraries were transduced into autologous immortalized B cells, followed by selection with puromycin. The cytotoxic potential of expanded patient TIL was confirmed before neoantigen screens by measuring their capacity to degranulate. To this end, CD4^+^ and CD8^+^ TIL were polyclonally stimulated using CD3/CD28 Dynabeads in T cell medium in the presence of Golgistop (BD Biosciences) and an antibody against CD107 for 12 h. Following incubation, cells were stained with IR-Dye and an anti-CD4 antibody and analyzed by flow cytometry. Neoantigen screens were subsequently performed by incubating library-expressing B cells in duplicate with patient T cells at a T cell:B cell ratio of 5:1. Library-transduced B cells cultured in the absence of patient T cells served as a negative control. After 72 h of coincubation, cells were processed as described above.

### Sequence analysis

Initial sequence quality profiles were quantified by FastQC and demultiplexed using fastq-multx (ea-utils) with one mismatch allowed. Vector sequences were trimmed from sequence reads using fastq-mcf (ea-utils) against the UniVec database, and samples were subsequently quality filtered using cutadapt. The unique 12 nt barcodes that were added to individual minigene sequences were extracted using seqkit and mapped using Bowtie2 with no multimatched hits allowed. For the ITO66 neoantigen screen, high-quality reads were mapped against the full minigene sequences of the patient library using BBMap with ambiguously mapped reads removed and only perfect mappings allowed. Per sample count tables were differentially compared and normalized using DESeq2. Minigenes with an average abundance below the fourth percentile and a coefficient of variation greater than one across the two internal replicates were removed from analyses. Statistical testing was performed using the DESeq2 Wald test and log-fold change cut-off of 0.25. Tumor variants were defined as screen hits when at least one of the duplicate mutant sequences, but neither of the corresponding WT-encoding minigenes, had an false discovery rate-corrected *P* value less than 0.2 and a log_2_ fold change of less than −0.5. All data analysis was performed using R and visualized using the ggplot2 package.

To validate patient neoantigens identified in screens, minigenes encoding the screen hits, as well as their WT counterparts, were synthesized as individual gBlocks (IDT), cloned into the CD74 signal sequence-modified pMSCV-puroR-mCherry vector and transduced into immortalized patient B cells. Following selection with puromycin, minigene-transduced B cells were cocultured with expanded patient CD4^+^ or CD8^+^ TIL for 48 h in T cell medium with 30 U ml^−1^ IL-2 and T cell activation was assessed by measuring IFNγ levels in the culture supernatant using the Cytometric Bead Array kit (BD Biosciences). Reactivity towards neoantigens was considered confirmed when T cells secreted at least twofold more IFNγ in response to the mutant sequence compared with the WT control sequence.

### NKIRTIL063 TMG screen

The number of tumor variants of patient NKIRTIL063 selected for TMG screening was reasonably limited to 200 (of a total of 685 nonsynonymous expressed mutations). Mutations were selected by first including the 25 most clonal mutations (based on variant allele frequency), followed by including mutations with highest gene expression up to a total of 200 tumor variants. TMG constructs were designed to encode ten variant-encoding minigenes (93 nt each) in which the mutant codon was flanked by 45 nt of nonmutant gene sequence on either side. Codon-optimized sequences were synthesized (Twist Biosciences) and subcloned into the CD74-modified pMSCV-puroR-mCherry retroviral vector. NKIRTIL063 immortalized B cells were transduced with TMG constructs and selected to more than 80% purity with 5 μg ml^−1^ puromycin. Next, TMG-expressing B cells were cocultured with NKIRTIL063 CD4^+^ or CD8^+^ TIL (from the same expansion cultures as used for the antigen discovery screen) at a ratio of 1:1 for 48 h in T cell medium with 30 U m^−1^ lL-2, and activation of T cells was determined by measuring IFNγ levels in the culture supernatant using the Cytometric Bead Array kit (BD Biosciences). To validate that the observed reactivity to selected TMG constructs was mediated by neoantigens identified using our neoantigen discovery screen, modified versions of these TMGs were designed such that exclusively the minigene that encoded the identified neoantigen was reverted to its WT sequence. Reactivity of NKIRTIL063 CD4^+^ or CD8^+^ TIL to B cells transduced with these modified TMGs was subsequently assessed as above.

### In silico selection of neoantigen vaccine targets

The computational tool Vaxrank^31^ was used to rank tumor mutations of patients NKIRTIL063, NKIRTIL027 and ITO66 for use in a putative personalized cancer vaccine. Patient ITO34 was omitted from this analysis because RNA expression data were unavailable. HLA typing of patients was performed using OptiType for HLA-A, -B and -C alleles. The set of somatic variant calls and aligned RNA reads were used as input, with parameters set to a peptide length of 25, an epitope length of 8–11 and utilization of the MHCFlurry prediction algorithm. In line with ongoing clinical trials of personalized neoantigen-based vaccines^32^, the 20 top ranking predicted neoantigens were considered for putative neoantigen vaccines (Supplementary Table 2).

### Reporting summary

Further information on research design is available in the Nature Portfolio Reporting Summary linked to this article.

## Online content

Any methods, additional references, Nature Portfolio reporting summaries, source data, extended data, supplementary information, acknowledgements, peer review information; details of author contributions and competing interests; and statements of data and code availability are available at 10.1038/s41587-022-01547-0.

## Supplementary information

Supplementary Information Supplementary Figs. 1–10.

Reporting Summary

Supplementary Table 1 Model antigen library and Vaxrank output.

## Data Availability

DNA sequencing data of antigen discovery screens have been deposited in the NCBI Sequence Read Archive under accession code PRJNA884260 (ref. ^30^). Protein sequences of genes encoding known human nonmutated cancer regression antigens, as well as selected viral genes were collected from the Uniprot database (https://www.uniprot.org/).

## Code availability

Scripts used for analyzing sequencing data from antigen discovery screens are available at https://github.com/twbattaglia/amplicon-nf (ref. ^33^). Script output for the presented analyses is available at https://github.com/twbattaglia/HANSolo-manuscript (ref. ^34^).
