# Supplementary material for: Identification of patient-specific CD4+ and CD8+ T cell neoantigens through HLA-unbiased genetic screens
Source: Nat Biotechnol. 2023 Jan 2;41(6):783–7. doi: 10.1038/s41587-022-01547-0 (PMC10264241; doi:10.1038/s41587-022-01547-0)
Supplement: Supplementary file 1 — Supplementary Figs. 1–10. [file 41587_2022_1547_MOESM1_ESM.pdf]

nature biotechnology

Brief Communication

https://doi.org/10.1038/s41587-022-01547-0

Identification of patient-specific CD4+ and CD8+ T cell neoantigens through HLA-unbiased genetic screens

In the format provided by the authors and unedited

Supplementary Information

**Supplementary Item**	**Title**
Supplementary Figure 1	Antigen specificity of $CDK4_{R>L}$- and $MART1_{26-35}$-specific model TCRs.
Supplementary Figure 2	Optimization of genetic screening conditions.
Supplementary Figure 3	Sensitivity analysis of genetic screens in the context of a diverse TCR repertoire.
Supplementary Figure 4	Generation of universal antigen expression system and antigen specificity of MHC class II-restricted model TCRs.
Supplementary Figure 5	Sensitivity of MHC class II-restricted neoantigen screens in the context of diverse TCR repertoires.
Supplementary Figure 6	NKIRTIL063 $CD4^+$ and $CD8^+$ TIL cytotoxicity and validation of reactivity to selected tumor variants.
Supplementary Figure 7	NKIRTIL027 $CD4^+$ and $CD8^+$ TIL cytotoxicity and reactivity to selected screen hits.
Supplementary Figure 8	ITO34 $CD4^+$ and $CD8^+$ TIL cytotoxicity and $CD4^+$ TIL neoantigen screen.
Supplementary Figure 9	ITO66 neoantigen screen using tumor organoid-induced PBMCs.
Supplementary Figure 10	Workflow for personalized neoantigen discovery using HLA-unbiased genetic screens.

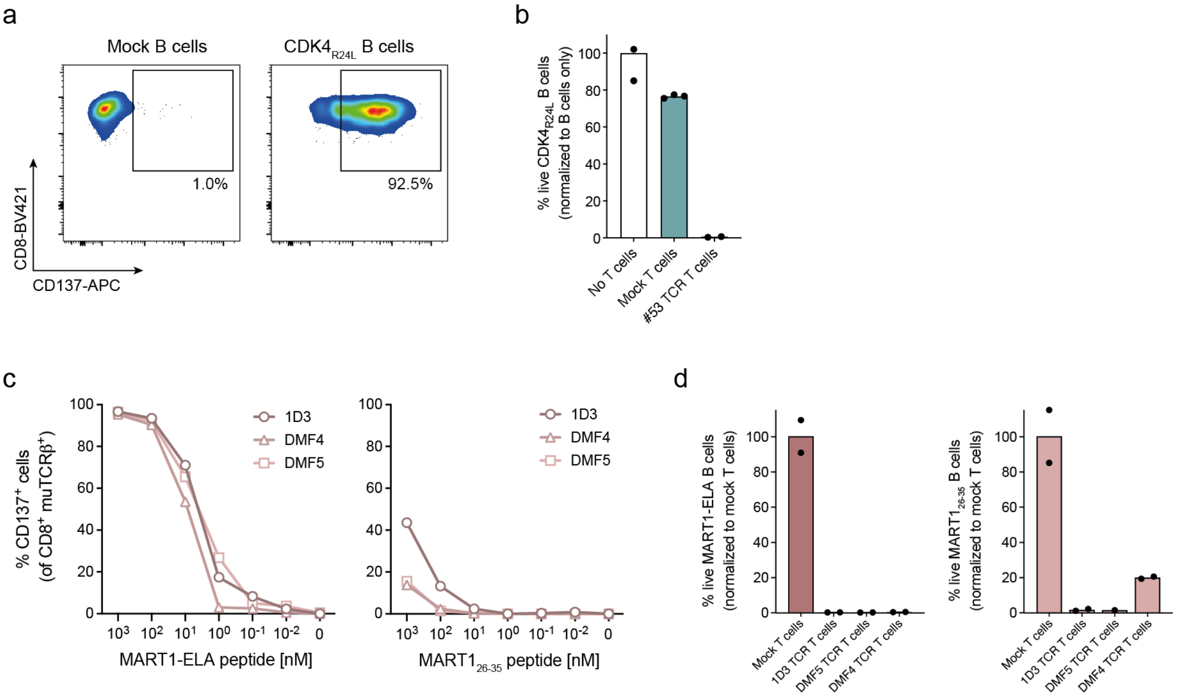

Supplementary Figure 1. Antigen specificity of CDK4$_{R>L}$- and MART1$_{26\text{-}35}$-specific model TCRs. (**A**) Donor CD8$^+$ T cells were transduced with the CDK4$_{R>L}$-specific TCR #53 and cocultured with HLA-A*02:01$^+$ B cells transduced with a CDK4$_{R>L}$-encoding minigene. Mock-transduced B cells served as negative control. T cell activation was assessed by measuring CD137 expression using flow cytometry. (**B**) Quantification of killing of B cells expressing a CDK4$_{R>L}$ minigene after a 72 hour co-culture with TCR #53-transduced donor CD8$^+$ T cells. Incubation of B cells in the presence of mock-transduced CD8$^+$ T cells served as a negative control, and data were normalized to the fraction of live B cells that was measured in the absence of T cells. Dots represent technical replicates. (**C**) Donor CD8$^+$ T cells were transduced with the MART1$_{26\text{-}35}$-specific DMF4, DMF5, or 1D3 TCR and cocultured with HLA-A*02:01$^+$ B cells pulsed with the indicated concentrations of MART1-ELA (left panel) or MART1$_{26\text{-}35}$ (right panel) peptide. T cell activation was evaluated by analysis of CD137 expression using flow cytometry. (**D**) Quantification of killing of B cells expressing either a MART1-ELA (left panel) or MART1$_{26\text{-}35}$ (right panel) minigene after a 72 hour co-culture with CD8$^+$ T cells expressing the 1D3, DMF4 or DMF5 TCR. Data were normalized to the fraction of live B cells that was measured in the presence of mock-transduced T cells. Dots represent technical replicates.

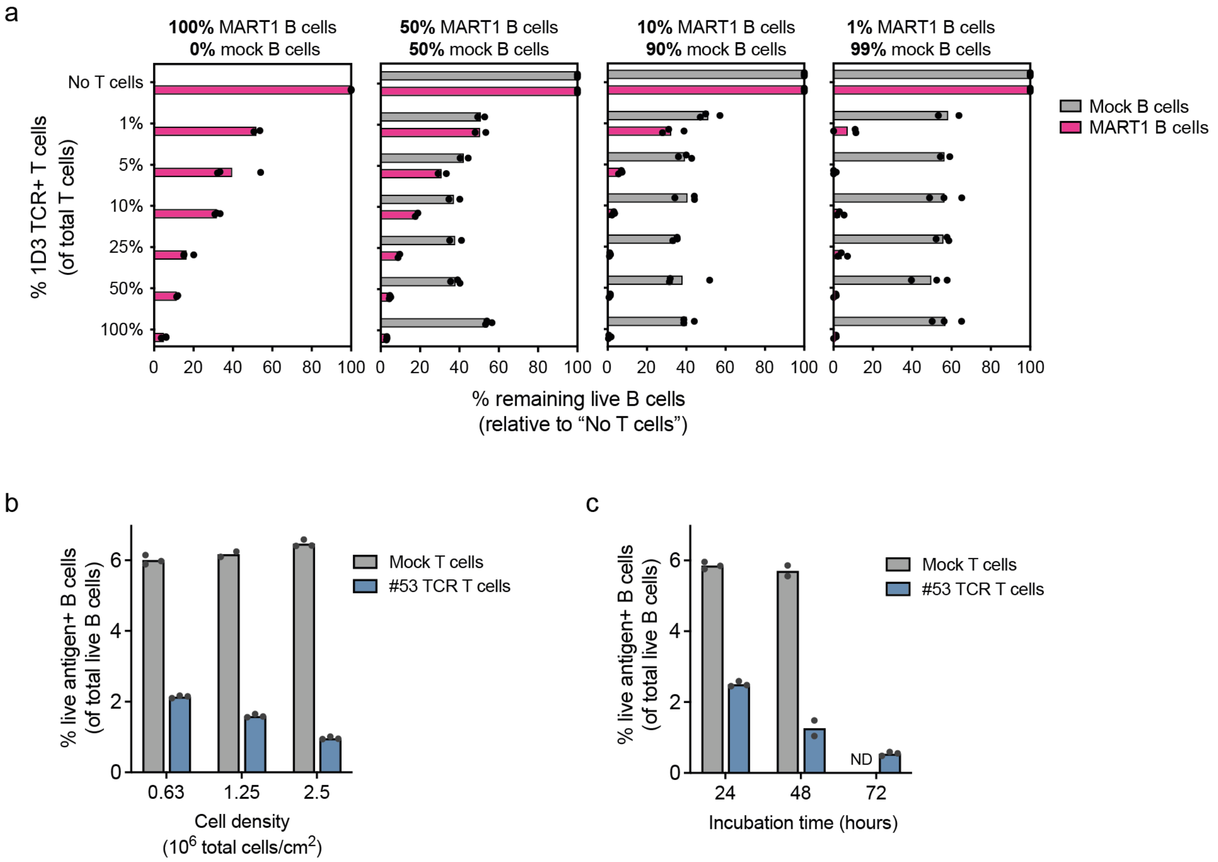

Supplementary Figure 2. Optimization of genetic screening conditions. (**A**) Donor CD8$^+$ T cells were transduced with the MART1$_{26\text{-}35}$-specific 1D3 TCR and mixed with mock-transduced T cells such that 1D3 TCR T cells comprised the indicated fractions of all T cells. Resulting T cell populations were incubated at a 5:1 E:T ratio with MART1-ELA minigene-transduced HLA-A*02:01$^+$ B cells that were mixed with mock-transduced B cells to the indicated fraction of all B cells. After 48 hours of incubation, B cell killing was quantified by flow cytometry. Data were normalized to the fraction of live B cells that was measured in the absence of T cells. Dots represent technical replicates. (**B**) CDK4$_{R>L}$ minigene-transduced B cells were mixed with mock-transduced B cells at a ratio of approximately 1:20, and incubated with CD8$^+$ T cells expressing the CDK4$_{R>L}$-specific #53 TCR at the indicated total cell densities, at an E:T ratio of 5:1. B cell killing was assessed using flow cytometry, and compared to incubation of B cell mixtures in the presence of mock-transduced CD8$^+$ T cells. Dots represent technical replicates. (**C**) CDK4$_{R>L}$ minigene-transduced B cells were mixed with mock-transduced B cells as in (B), and incubated with CD8$^+$ #53 TCR T cells for the indicated duration, at an E:T ratio of 5:1 and total cell density of 2.5 x 10^6 cells/cm^2. Incubation of B cells in the presence of mock-transduced CD8$^+$ T cells served as negative control. B cell killing was quantified using flow cytometry. N.D. is not determined. Dots represent technical replicates.

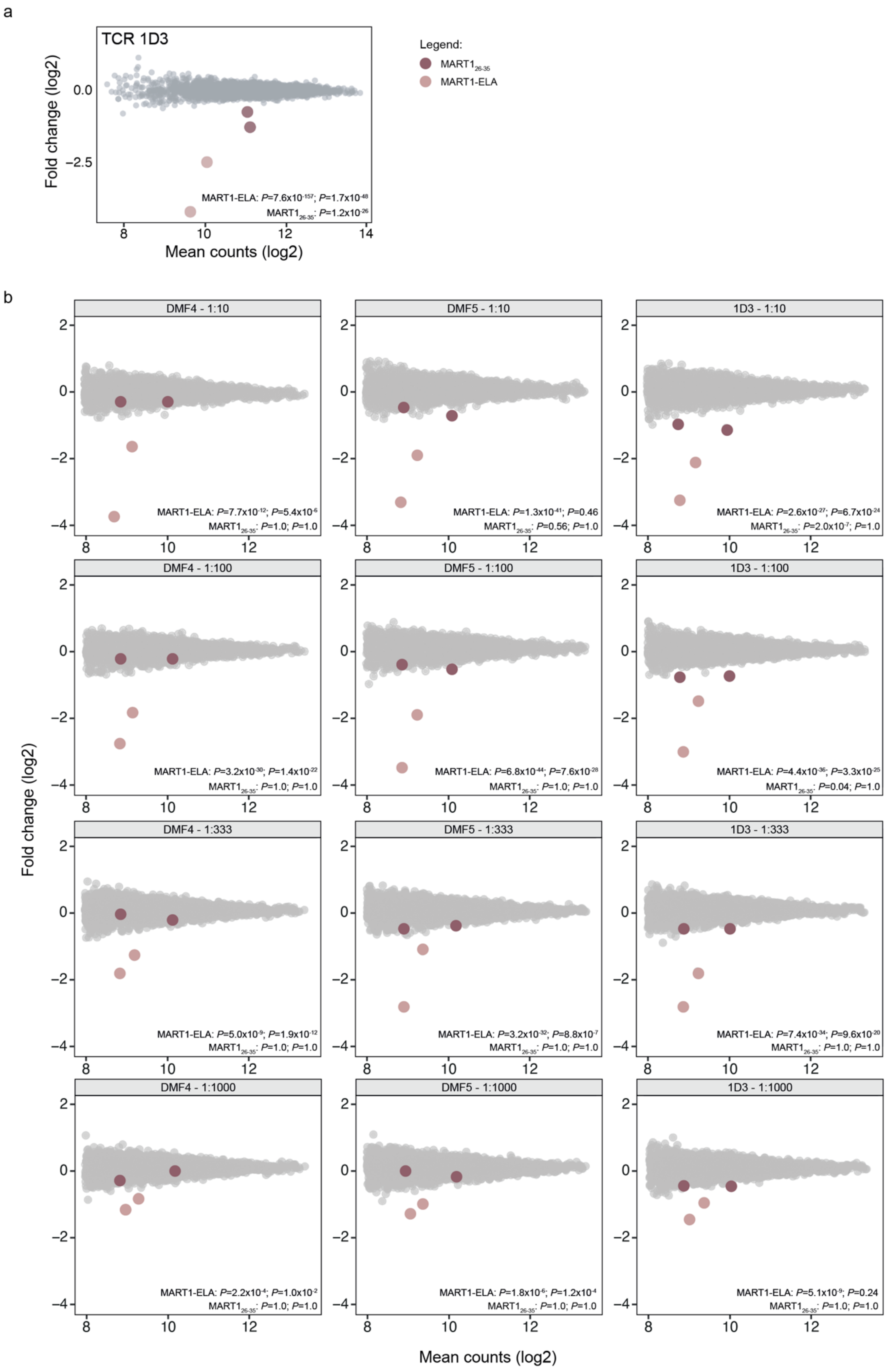

Supplementary Figure 3. Sensitivity analysis of genetic screens in the context of a diverse TCR repertoire. (**A**) Genetic screen using CD8^{+} T cells expressing the MART1$_{26\text{-}35}$-specific 1D3 TCR and HLA-A*02:01^{+} B cells expressing the model epitope library (n=4,764 minigenes). Dots represent individual

minigenes. Fold change, defined as relative minigene abundance when exposed to either 1D3 TCR T cells or mock T cells, and mean normalized read counts are plotted for each individual minigene. Minigenes encoding the MART1-ELA and native $MART1_{26\text{-}35}$ epitopes are highlighted. P-values were generated using DESeq2's Wald test (one-sided) and adjusted for multiple comparisons. Data depicted here and in Fig. 1C were generated in independent experiments. (**B**) $CD8^+$ T cells expressing either the DMF4, DMF5 or 1D3 TCR were mixed with mock-transduced T cells to reach the indicated frequencies, and were incubated with HLA-A*02:01$^+$ B cells expressing the model epitope library (n=4,764 minigenes). Data are plotted as in (A), with fold change reflecting relative minigene abundance when exposed to DMF4 or DMF5 TCR T cells as compared to mock T cells. Data depicted were generated within a single experiment.

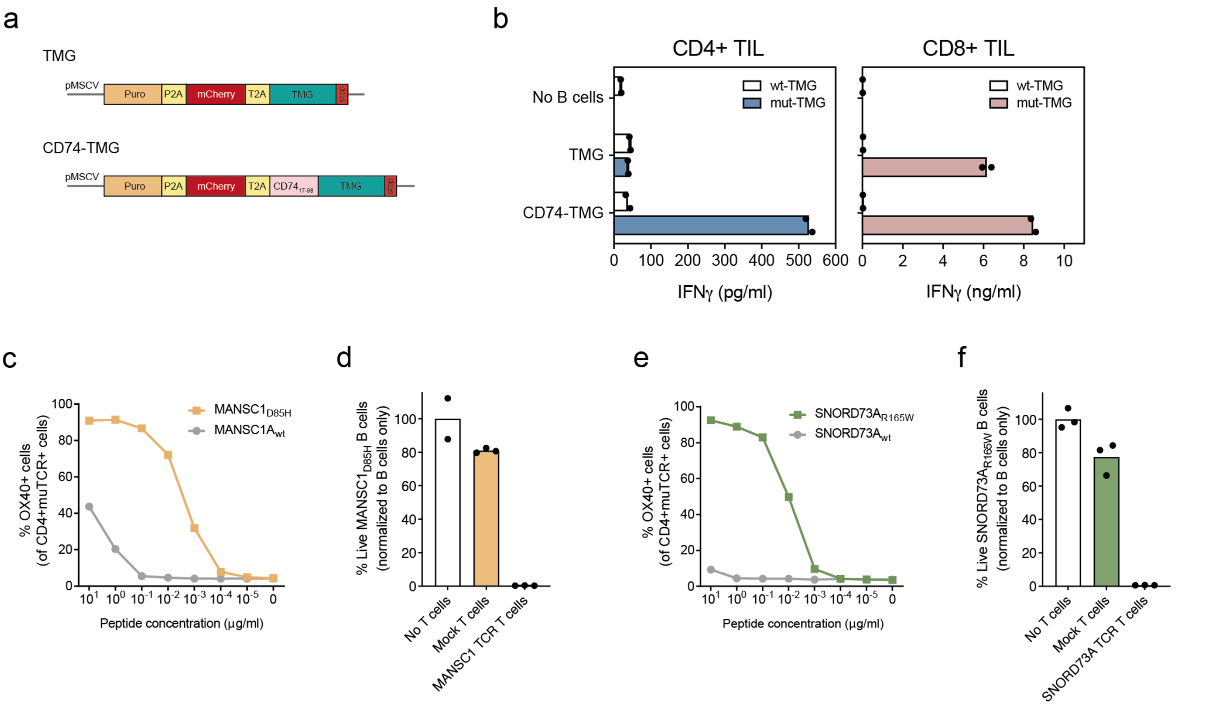

Supplementary Figure 4. Generation of universal antigen expression system and antigen specificity of MHC class II-restricted model TCRs. (**A**) Schematic overview of the incorporation of the CD74 sorting signal in the pMSCV vector to enable processing and presentation of minigene products through both MHC class I and class II pathways. (**B**) Patient NKIRTIL027 *in vitro*-expanded CD4$^+$ and CD8$^+$ TIL were incubated with autologous immortalized B cells that were transduced with the indicated variants of a TMG construct encoding two previously identified neoantigens recognized by either NKIRTIL027 CD4$^+$ or CD8$^+$ TIL (LEMD2$_{P>L}$ and TTC37$_{A>V}$, respectively). T cell activation was assessed by analysis of IFNγ secretion. Data represent mean IFNγ levels of technical replicates. (**C-F**) Characterization of patient-derived MANSC1$_{D>H}$- and SNORD73A$_{R>W}$-specific TCRs. TCRs were expressed in donor CD4$^+$ T cells and resulting cells were incubated with patient-matched B cells pulsed with the indicated concentrations of MANSC1$_{D>H}$ (C) or SNORD73A$_{R>W}$ (E) peptides, or the respective wild-type control peptides. T cell activation was assessed by analysis of OX40 surface expression. Data depict mean OX40 levels of technical replicates. Killing of B cells expressing MANSC1$_{D>H}$- (D) or SNORD73A$_{R>W}$ (F) minigenes was quantified after a 72 hour co-culture with donor CD4$^+$ T cells transduced to express the indicated TCRs. Incubation of B cells in the presence of mock-transduced T cells served as a negative control, and data were normalized to the fraction of live B cells that were detected in the absence of T cells. Dots represent technical replicates.

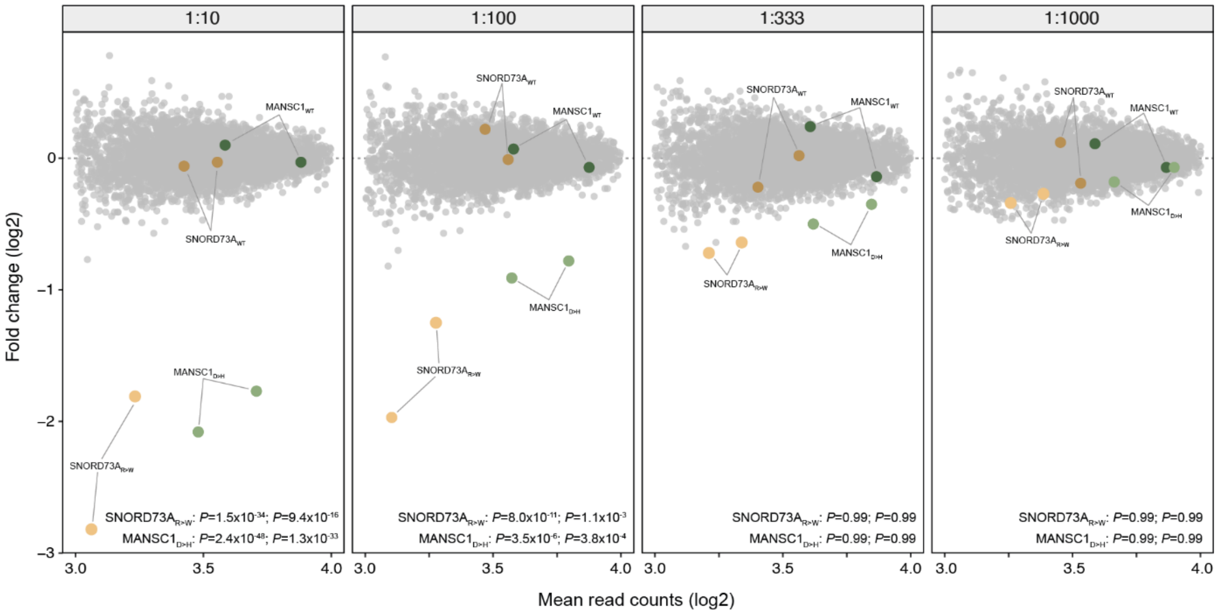

Supplementary Figure 5. Sensitivity of MHC class II-restricted neoantigen screens in the context of diverse TCR repertoires. CD4+ T cells expressing either the MANSC1$_{D>H}$-specific or SNORD73A$_{R>W}$-specific TCR were mixed with mock-transduced T cells such that MANSC1$_{D>H}$- or SNORD73A$_{R>W}$-TCR T cells comprised the indicated fraction of all T cells, and T cell mixtures were then incubated with matched B cells that were transduced with the model antigen library (n=4,764 minigenes). Dots represent individual minigenes. Fold change, defined as relative minigene abundance when exposed to MANSC1 and SNORD73A TCR T cells as compared to mock T cells, and mean normalized read counts are plotted for each individual minigene. Minigenes encoding the mutant and wild-type MANSC1 and SNORD73A epitopes are highlighted. P-values were generated using DESeq2's Wald test (one-sided) and adjusted for multiple comparisons.

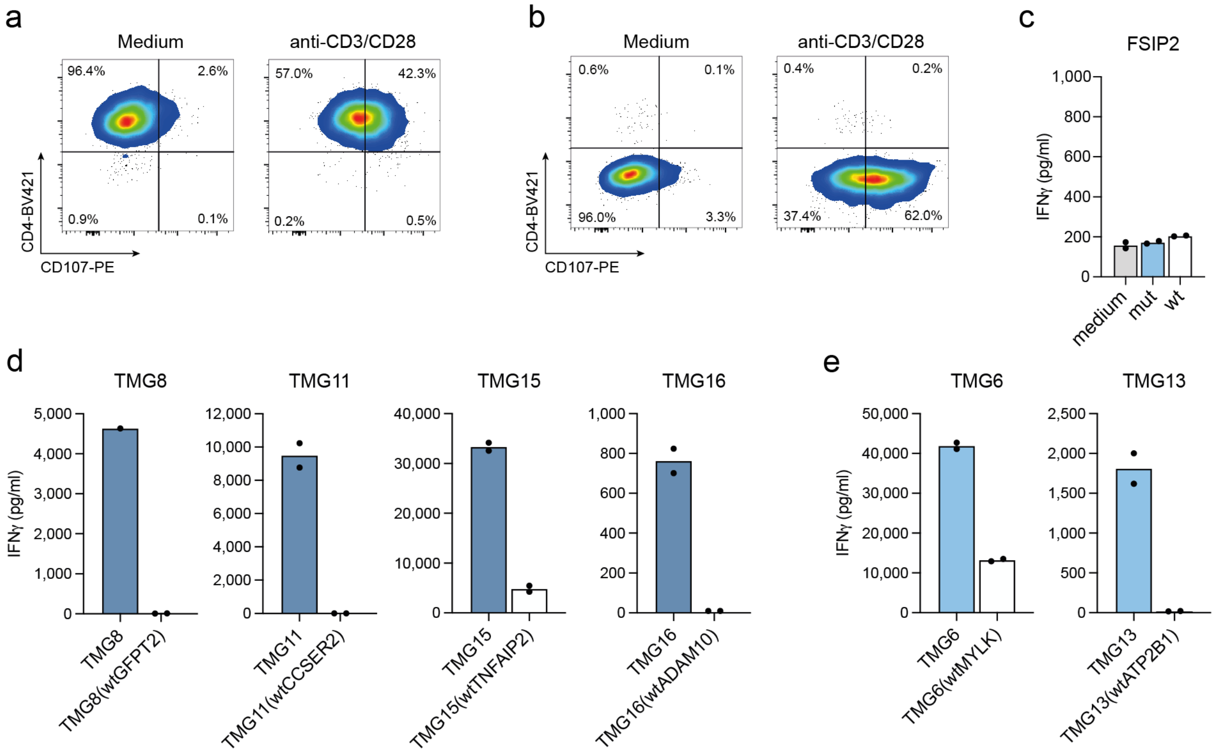

Supplementary Figure 6. NKIRTIL063 CD4⁺ and CD8⁺ TIL cytotoxicity and validation of reactivity to selected tumor variants. (**A,B**) *In vitro*-expanded NKIRTIL063 CD4⁺ (A) and CD8⁺ (B) TIL were stimulated with anti-CD3/CD28 antibody-coated beads in the presence of monensin and PE-conjugated anti-CD107 antibody, and CD107 surface expression was measured by flow cytometry. (**C**) Lack of reactivity of NKIRTIL063 CD4⁺ TIL towards mutated FSIP2. CD4⁺ TIL were incubated with patient B cells expressing either the screen hit FSIP2 or its wild-type counterpart, and T cell activation was assessed by analysis of IFNγ levels. T cells cultured in the absence of B cells served as negative control. Dots represent technical replicates. (**D,E**) NKIRTIL063 B cells were transduced with the indicated TMGs or with the same TMGs in which exclusively the mutant sequence of the screen hit was reverted to its corresponding wild-type sequence. Resulting B cells were subsequently incubated with NKIRTIL063 CD8⁺ (D) or CD4⁺ TIL (E), and T cell reactivity was assessed by analysis of IFNγ secretion. Dots represent technical replicates.

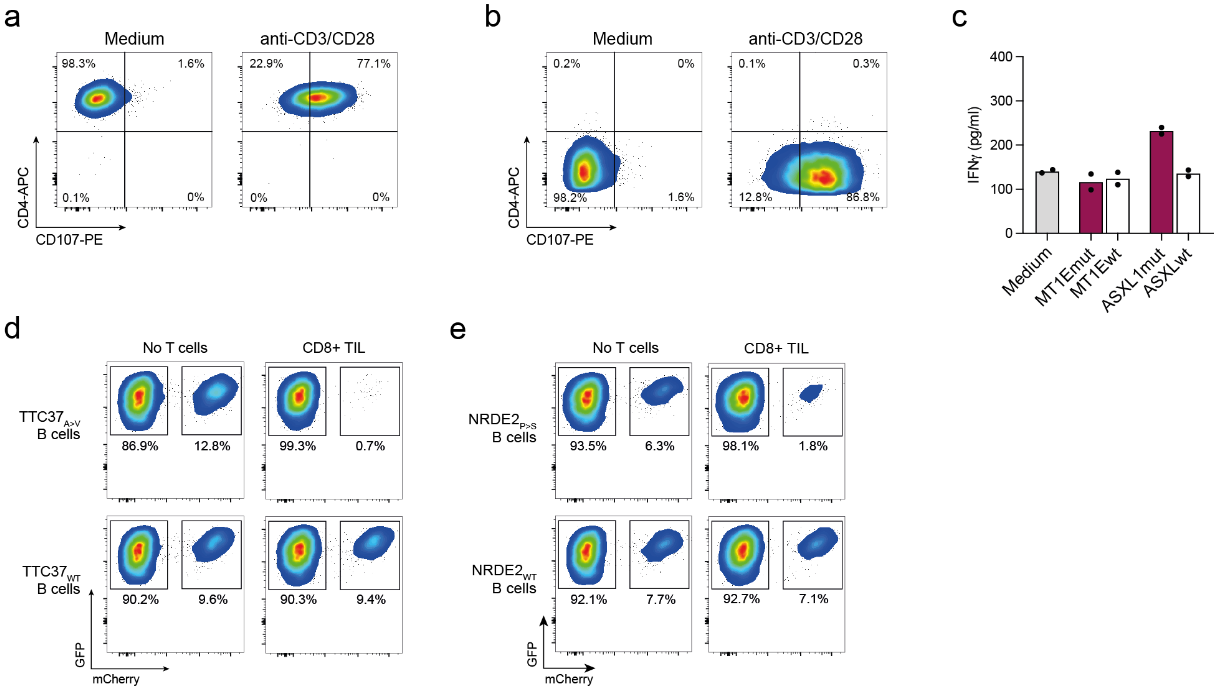

Supplementary Figure 7. NKIRTIL027 CD4$^+$ and CD8$^+$ TIL cytotoxicity and reactivity to selected screen hits. (**A,B**) *In vitro*-expanded NKIRTIL027 CD4$^+$ (A) and CD8$^+$ (B) TIL were stimulated with anti-CD3/CD28 antibody-coated beads in the presence of monensin and PE-conjugated anti-CD107 antibody, and CD107 surface expression was measured by flow cytometry. (**C**) Lack of reactivity of NKIRTIL027 CD8$^+$ TIL towards mutated MT1E and ASXL1. CD8$^+$ TIL were incubated with patient B cells expressing the indicated screen hits or their wild-type counterparts (see Fig. 2G), and T cell activation was assessed by analysis of IFNγ. T cells cultured in the absence of B cells served as negative control. Dots represent technical replicates. (**D,E**) NKIRTIL027 B cells transduced with minigenes encoding the screen hits $TTC37_{A>V}$ (D) or $NRDE2_{P>S}$ (E) or their respective wild-type counterparts were mixed with untransduced patient B cells and incubated with patient CD8$^+$ T cells. Incubation of B cells in the absence of T cells served as negative control. After 72 hours, the presence of minigene-expressing (GFP$^+$mCherry$^+$) B cells relative to untransduced (GFP$^+$mCherry$^-$) B cells was analysed by flow cytometry. Data depict representative flow cytometry plots.

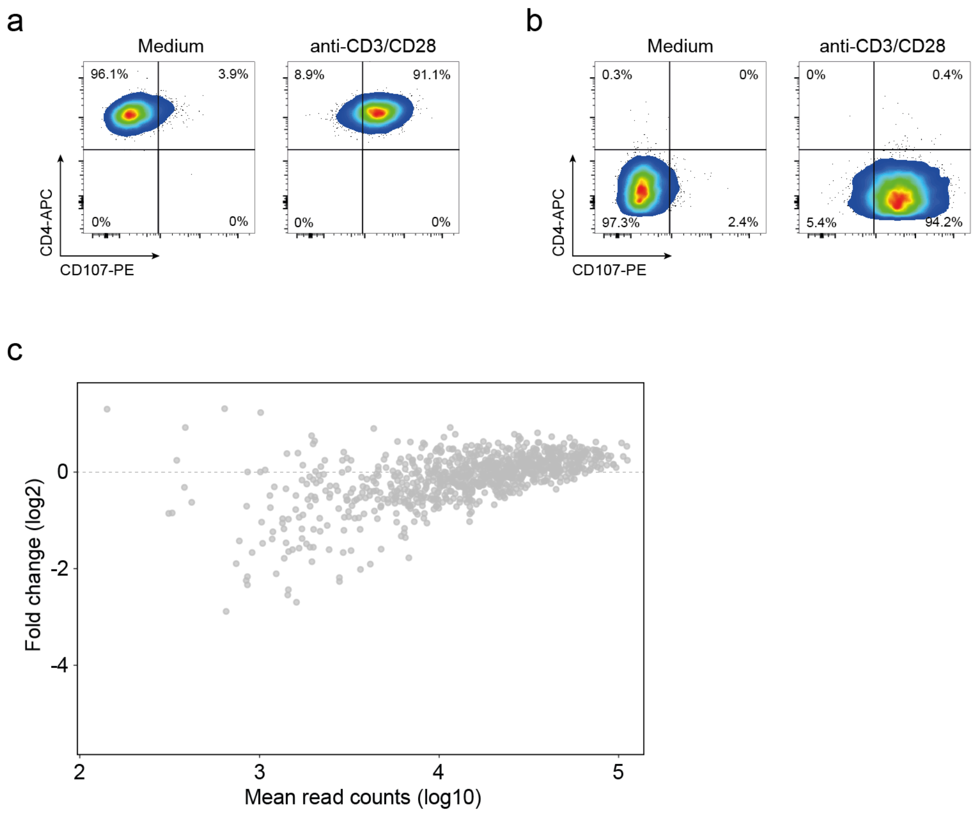

Supplementary Figure 8. ITO34 CD4⁺ and CD8⁺ TIL cytotoxicity and CD4⁺ TIL neoantigen screen. (**A,B**) *In vitro*-expanded ITO34 CD4⁺ (A) and CD8⁺ (B) TIL were stimulated with anti-CD3/CD28 antibody-coated beads in the presence of monensin and PE-conjugated anti-CD107 antibody, and CD107 surface expression was measured by flow cytometry. (**C**) Neoantigen specificities of patient ITO34 CD4⁺ TIL were evaluated by antigen discovery screen as in Fig. 2I.

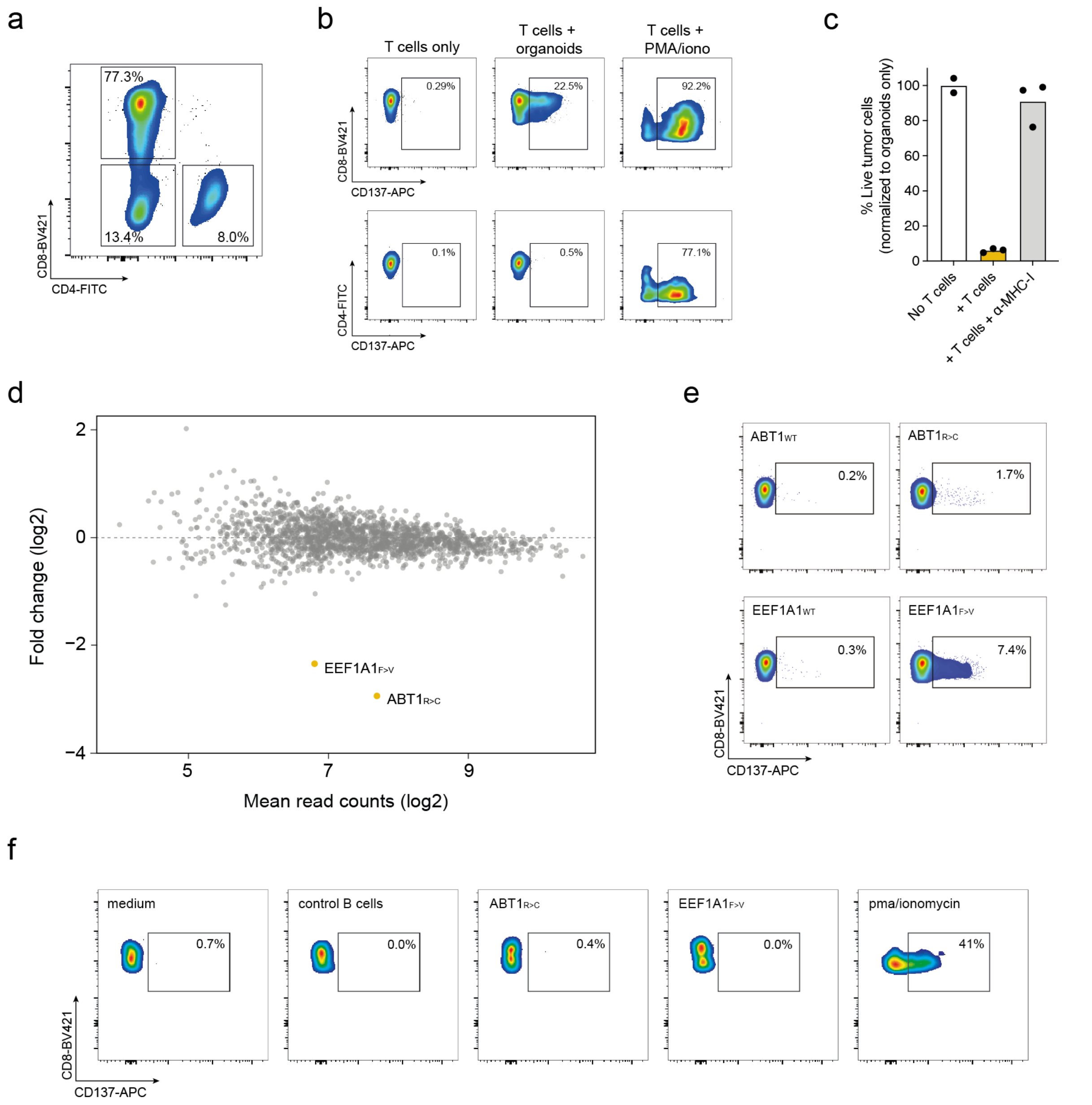

Supplementary Figure 9. ITO66 neoantigen screen using tumor organoid-induced PBMCs. (**A**) Tumor-specific T cells were obtained by co-incubation of PBMCs of patient ITO66 with matched tumor organoids for two weeks. The fractions of $CD4^+$, $CD8^+$ and $CD4^-CD8^-$ T cells were analyzed by flow cytometry. Depicted data are gated on live single $CD3^+$ cells. (**B**) Reactivity of organoid-induced patient PBMCs is restricted to $CD8^+$ T cells. Tumor-reactivity of the ITO66 cell product was assessed by incubation of cells with tumor organoids and analysis of CD137 surface expression on $CD8^+$ (top) and $CD4^+$ (bottom) cells. Data depict representative flow cytometry plots. (**C**) Quantification of patient PBMC cytotoxicity towards matched tumor organoids. Data were normalized to the fraction of live B cells that were detected in the absence of T cells. To assess the contribution of MHC class I-restricted T cells to organoid killing, patient PBMCs and organoids were co-incubated in the presence or absence of an MHC class I-blocking antibody. Dots represent technical replicates. (**D**) Patient ITO66 immortalized B cells were transduced with the patient mutanome library (n=1,834 minigenes) and screened using organoid-induced PBMCs. Fold change depicts relative minigene abundance in cultures with or without patient PBMCs. Screen hits are marked by colored dots. (**E**) Neoantigen hits

identified in (D) were validated by expression of EEF1A1$_{F>V}$, ABT1$_{R>C}$ or their respective wild-type counterparts as single minigenes in patient B cells and subsequent incubation of transduced B cells with organoid-induced patient PBMCs. CD8^{+} T cell activation was assessed by analysis of CD137 surface expression. Data depict representative flow cytometry plots. (**F**) Neoantigen reactivity among ITO66 PBMCs prior to co-incubation with patient tumor organoids was assessed by incubating patient PBMCs with matched B cells expressing neoantigen-encoding minigenes and measuring CD137 surface expression. PBMCs were derived from the same batch as used for the generation of tumor-specific T cells by incubation with tumor organoids. PMA and ionomycin stimulation of patient PBMCs served as positive control. Data depict representative flow cytometry plots.

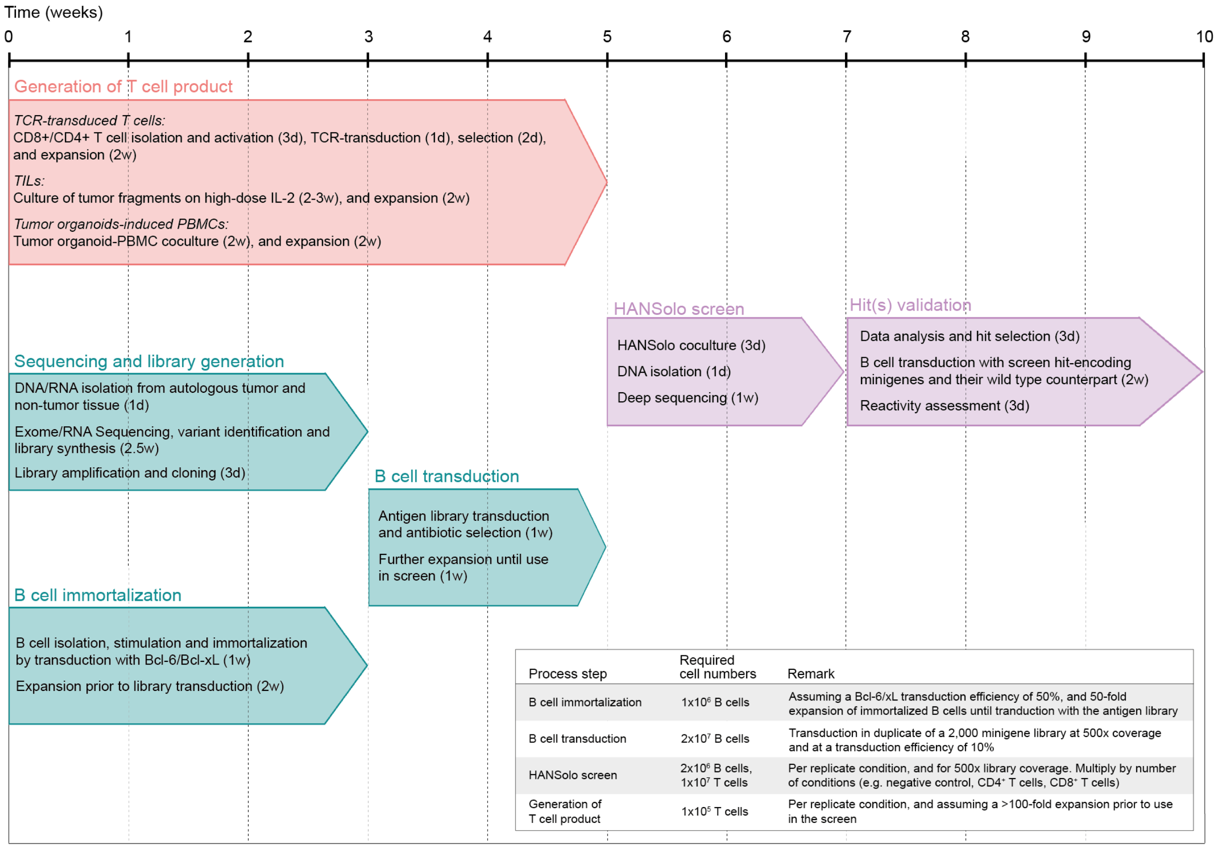

Process step	Required cell numbers	Remark
B cell immortalization	$1x10^6$ B cells	Assuming a Bcl-6/xL transduction efficiency of 50%, and 50-fold expansion of immortalized B cells until tranduction with the antigen library
B cell transduction	$2x10^7$ B cells	Transduction in duplicate of a 2,000 minigene library at 500x coverage and at a transduction efficiency of 10%
HANSolo screen	$2x10^6$ B cells, $1x10^7$ T cells	Per replicate condition, and for 500x library coverage. Multiply by number of conditions (e.g. negative control, $CD4^+$ T cells, $CD8^+$ T cells)
Generation of T cell product	$1x10^5$ T cells	Per replicate condition, and assuming a >100-fold expansion prior to use in the screen

Supplementary Figure 10. Workflow for personalized neoantigen discovery using HLA-unbiased genetic screens. The time required to complete each step is specified in weeks (w) or days (d). The box outlines the B and T cell numbers that are required to perform a neoantigen screen in the context of an example patient with 500 tumor mutations.
