# Supplementary material for: Identification of patient-specific CD4+ and CD8+ T cell neoantigens through HLA-unbiased genetic screens
Source: Nat Biotechnol. 2023 Jan 2;41(6):783–7. doi: 10.1038/s41587-022-01547-0 (PMC10264241; doi:10.1038/s41587-022-01547-0)
Supplement: Supplementary file 2 — Reporting Summary [file 41587_2022_1547_MOESM2_ESM.pdf]

nature research

Corresponding author(s): Wouter Scheper
Ton Schumacher
Emile Voest

Last updated by author(s): 27/9/22

Reporting Summary

Nature Research wishes to improve the reproducibility of the work that we publish. This form provides structure for consistency and transparency in reporting. For further information on Nature Research policies, see our Editorial Policies and the Editorial Policy Checklist.

Statistics

For all statistical analyses, confirm that the following items are present in the figure legend, table legend, main text, or Methods section.

n/a	Confirmed	
☐	☒	The exact sample size (*n*) for each experimental group/condition, given as a discrete number and unit of measurement
☒	☐	A statement on whether measurements were taken from distinct samples or whether the same sample was measured repeatedly
☐	☒	The statistical test(s) used AND whether they are one- or two-sided<br>*Only common tests should be described solely by name; describe more complex techniques in the Methods section.*
☒	☐	A description of all covariates tested
☐	☒	A description of any assumptions or corrections, such as tests of normality and adjustment for multiple comparisons
☐	☒	A full description of the statistical parameters including central tendency (e.g. means) or other basic estimates (e.g. regression coefficient) AND variation (e.g. standard deviation) or associated estimates of uncertainty (e.g. confidence intervals)
☐	☒	For null hypothesis testing, the test statistic (e.g. *F*, *t*, *r*) with confidence intervals, effect sizes, degrees of freedom and *P* value noted<br>*Give P values as exact values whenever suitable.*
☒	☐	For Bayesian analysis, information on the choice of priors and Markov chain Monte Carlo settings
☒	☐	For hierarchical and complex designs, identification of the appropriate level for tests and full reporting of outcomes
☒	☐	Estimates of effect sizes (e.g. Cohen's *d*, Pearson's *r*), indicating how they were calculated

Our web collection on statistics for biologists contains articles on many of the points above.

Software and code

Policy information about availability of computer code

Data collection

BD FACSDiva 8.0.2

Data analysis

Mutational detection: The bcbio-nextgen v1.2.9 Tumor/Normal workflow was used to call variants against genome build GRCh38 (Release 88). DNA was aligned using BWA v0.7.17, duplicates were marked with Picard MarkDuplicates v2.23.9 and low complexity regions were excluded. Somatic variants were called with MuTect2 v3.7-0 and germline variants were called with HaplotypeCaller v3.8. SnpSift v4.3p was used for variant annotation.

Gene expression: The bcbio-nextgen v1.2.9 RNAseq workflow was used to determine gene expression using Salmon v1.4.0. Gene fusions were determined using Arriba v2.1.0 after alignment with STAR v2.6.1d. For ITO66, RNA-reads were aligned using TopHat2 v2.1.0.

Library sequencing processing: FastQC v0.11.9 was used for sequence quality analysis, ea-utils v1.1.2.779 was used to remove adapters (fastq-multx) and vectors (fastq-mcf) from the UniVec database, cutadapt v3.7 for quality filtering, barcodes were extracted using seqkit v0.15.0 and read alignment was performed using Bowtie2 v2.4.2 and BBMap v38.90. A reproducible pipeline can be found at https://github.com/twbattaglia/amplicon-nf .

Data analysis: Count data was imported into R v4.1.3, visualized with ggplot2 v3.3.3 and statistical testing was performed using DESeq2 v1.34. A reproducible notebook of the analysis can be found at https://github.com/twbattaglia/HANSolo-manuscript .

Neoantigen prediction: OptiType v1.3.4 and BBMap v38.87 were used to gather HLA typing information, RNAseq data was aligned with TopHat v2.1.0 against genome build GRCh38 (Release 77) or STAR v2.6.1d and Vaxrank v1.4.0 was used determine personal cancer vaccines, using MHCFlurry 2.0 to predict peptide-MHC affinity.

Flow cytometry: Flowjo (version 10.0.7)

Data processing and plotting: Graphad Prism (version 9.3.1)

For manuscripts utilizing custom algorithms or software that are central to the research but not yet described in published literature, software must be made available to editors and reviewers. We strongly encourage code deposition in a community repository (e.g. GitHub). See the Nature Research guidelines for submitting code & software for further information.

Data

Policy information about availability of data

All manuscripts must include a data availability statement. This statement should provide the following information, where applicable:

- Accession codes, unique identifiers, or web links for publicly available datasets
- A list of figures that have associated raw data
- A description of any restrictions on data availability

DNA sequencing data of antigen discovery screens have been deposited in the NCBI Sequence Read Archive under accession code PRJNA884260.

To design the model antigen library, protein sequences of genes encoding known human non-mutated cancer regression antigens, as well as selected viral genes were collected from the Uniprot database (www.uniprot.org).

Field-specific reporting

Please select the one below that is the best fit for your research. If you are not sure, read the appropriate sections before making your selection.

☒ Life sciences ☐ Behavioural & social sciences ☐ Ecological, evolutionary & environmental sciences

For a reference copy of the document with all sections, see nature.com/documents/nr-reporting-summary-flat.pdf

Life sciences study design

All studies must disclose on these points even when the disclosure is negative.

Sample size	In line with the proof-of-concept nature of the manuscript, validation of the developed antigen discovery screening method was performed in a small cohort of four cancer patients. No conclusions are drawn regarding a broader patient populations based on these data.
Data exclusions	No data were excluded from the analyses.
Replication	For genetic antigen discovery screens, each screening condition was tested in duplicate, and each epitope was encoded twice in minigene libraries (resulting in four measurements per minigene-encoded epitope). Antigen screens using the model antigen library were performed at least twice, Figure 1 shows results from representative experiments. Due to time considerations, patient neoantigen screens were performed once. T cell activation experiments to validate neoantigen hits from patient screens were performed once.
Randomization	No experimental groups were employed in this work, and sample allocation was therefore not possible nor relevant.
Blinding	No experimental groups were employed in this work, and blinding was therefore not possible nor relevant.

Reporting for specific materials, systems and methods

We require information from authors about some types of materials, experimental systems and methods used in many studies. Here, indicate whether each material, system or method listed is relevant to your study. If you are not sure if a list item applies to your research, read the appropriate section before selecting a response.

Materials & experimental systems

n/a	Involved in the study	
☐	☒	Antibodies
☐	☒	Eukaryotic cell lines
☒	☐	Palaeontology and archaeology
☒	☐	Animals and other organisms
☐	☒	Human research participants
☒	☐	Clinical data
☒	☐	Dual use research of concern

Methods

n/a	Involved in the study	
☒	☐	ChIP-seq
☐	☒	Flow cytometry
☒	☐	MRI-based neuroimaging

Antibodies

Antibodies used	Mouse anti-human CD28, clone CD28.2 (eBioscience, cat. no. 16-0289-81; 5 µg/ml final concentration)<br>Mouse anti-human CD3, PerCP-Cy5.5-conjugated, clone SK7 (BD, cat. no. 332771; dilution 1:20)

	Mouse anti-human CD4, FITC-conjugated, clone RPA-T4 (BD, cat. no. 555346; dilution 1:20)<br>Mouse anti-human CD4, APC-conjugated, clone RPA-T4 (BD, cat. no. 555349; dilution 1:30)<br>Mouse anti-human CD4, BV421-conjugated, clone SK3 (Biolegend, cat. no. 344632; dilution 1:100)<br>Mouse anti-human CD8, BV421-conjugated, clone RPA-T8 (BD, cat. no. 562429; dilution 1:50)<br>Mouse anti-human CD137, APC-conjugated, clone 4B4-1 (BD, cat. no. 550890; dilution 1:30)<br>Mouse anti-human CD137, BV421-conjugated, clone 4B4-1 (Biolegend, cat. no. 309819; dilution 1:200)<br>Mouse anti-human OX40, PE-Cy-conjugated, clone Ber-ACT35 (Biolegend, cat. no. 350012; dilution 1:50)<br>Mouse anti-human CD107a, PE-conjugated, clone H4A3 (BD, cat. no. 555801; dilution 1:50)<br>Mouse anti-human CD14, APC-H7-conjugated, clone MoP9 (BD, cat. no. 560180; dilution 1:100)<br>Mouse anti-human CD16, APC-H7-conjugated, clone 3G8 (BD, cat. no. 560715; dilution 1:100)<br>Mouse anti-human CD19, FITC-conjugated. clone 4G7 (BD, cat. no. 345776; dilution 1:30)<br>Hamster anti-mouse TCRbeta constant domain, PE-conjugated, clone H57-597 (BD, cat. no. 561081; dilution 1:150)
Validation	All antibodies were validated by antibody suppliers, as detailed on the suppliers' websites. The species specificity of the anti-mouse TCRbeta constant domain antibody was furthermore validated for our application using human T cells that were transduced (where staining is present) or not transduced (where staining is absent) with murinized TCRs. Validation of antibodies against T cell activation markers (CD137, OX40 and CD107) furthermore included upregulation of markers on PHA-stimulated, but not unstimulated human PBMCs.<br><br>Mouse anti-human CD28: https://www.thermofisher.com/antibody/product/CD28-Antibody-clone-CD28-2-Monoclonal/16-0289-81. Validated from the supplier's website and the references reported there.<br>Mouse anti-human CD3-PerCP-Cy5.5: https://www.bdbiosciences.com/en-gb/products/reagents/flow-cytometry-reagents/clinical-diagnostics/single-color-antibodies-asr-ivd-ce-ivd/cd3-percp-cy-5-5.332771. Validated from the supplier's website and the references reported there.<br>Mouse anti-human CD4-FITC: https://www.bdbiosciences.com/en-gb/products/reagents/flow-cytometry-reagents/research-reagents/single-color-antibodies-ruo/fitc-mouse-anti-human-cd4.555346. Validated from the supplier's website and the references reported there.<br>Mouse anti-human CD4-APC: https://www.bdbiosciences.com/en-gb/products/reagents/flow-cytometry-reagents/research-reagents/single-color-antibodies-ruo/apc-mouse-anti-human-cd4.555349. Validated from the supplier's website and the references reported there.<br>Mouse anti-human CD4-BV421: https://www.biolegend.com/nl-nl/products/brilliant-violet-421-anti-human-cd4-antibody-12068. Validated from the supplier's website and the references reported there.<br>Mouse anti-human CD8-BV421: https://www.bdbiosciences.com/en-gb/products/reagents/flow-cytometry-reagents/research-reagents/single-color-antibodies-ruo/bv421-mouse-anti-human-cd8.562429. Validated from the supplier's website and the references reported there.<br>Mouse anti-human CD137-APC: https://www.bdbiosciences.com/en-gb/products/reagents/flow-cytometry-reagents/research-reagents/single-color-antibodies-ruo/apc-mouse-anti-human-cd137.550890. Validated from the supplier's website and the references reported there.<br>Mouse anti-human CD137-BV421: https://www.biolegend.com/en-us/products/brilliant-violet-421-anti-human-cd137-4-1bb-antibody-7211. Validated from the supplier's website and the references reported there.<br>Mouse anti-human OX40-PE-Cy7: https://www.biolegend.com/en-us/products/pe-cyanine7-anti-human-cd134-ox40-antibody-7234. Validated from the supplier's website and the references reported there.<br>Mouse anti-human CD107a-PE: https://www.bdbiosciences.com/en-gb/products/reagents/flow-cytometry-reagents/research-reagents/single-color-antibodies-ruo/pe-mouse-anti-human-cd107a.555801. Validated from the supplier's website and the references reported there.<br>Mouse anti-human CD14-APC-H7: https://www.bdbiosciences.com/en-gb/products/reagents/flow-cytometry-reagents/research-reagents/single-color-antibodies-ruo/apc-h7-mouse-anti-human-cd14.560180. Validated from the supplier's website and the references reported there.<br>Mouse anti-human CD16-APC-H7: https://www.bdbiosciences.com/en-gb/products/reagents/flow-cytometry-reagents/research-reagents/single-color-antibodies-ruo/apc-h7-mouse-anti-human-cd16.560715. Validated from the supplier's website and the references reported there.<br>Mouse anti-human CD19-FITC: https://www.bdbiosciences.com/en-gb/products/reagents/flow-cytometry-reagents/clinical-diagnostics/single-color-antibodies-asr-ivd-ce-ivd/cd19-fitc.345776. Validated from the supplier's website and the references reported there.<br>Hamster anti-mouse TCRbeta constant domain PE: https://www.bdbiosciences.com/en-gb/products/reagents/flow-cytometry-reagents/research-reagents/single-color-antibodies-ruo/pe-hamster-anti-mouse-tcr-chain.561081

Eukaryotic cell lines

Policy information about cell lines

Cell line source(s)	OVC21 B cells, NKIRTIL017 B cells, NKIRTIL027 B cells, NKIRTIL063 B cells, ITO34 B cells, ITO66 B cells and ITO66 tumor organoids were generated from patients treated at the Netherlands Cancer Institute.<br>The FLY-RD18 cell line was commercially obtained from Sigma.
Authentication	Patient-derived B cell and organoid lines were authenticated by SNParray on germline DNA and cell line- and/or organoids-derived DNA.<br>FLY-RD18 cells were validated morphologically and by their capacity to produce retrovirus.
Mycoplasma contamination	Cell lines were tested negative for Mycoplasma contamination.
Commonly misidentified lines<br>(See ICLAC register)	No commonly misidentified cell lines were used.

Human research participants

Policy information about studies involving human research participants

Population characteristics	Patient: NKIRTIL027; sex: female; age: 44; diagnosis: melanoma; tumor location: lymph node metastasis; stage: IV; genotypic information: BRAF(V600E).<br><br>Patient: NKIRTIL063; sex: female; age: 57; diagnosis: melanoma; tumor location: subcutaneous metastasis; stage: IV; genotypic information: NRAS(Q61K),<br><br>Patient: ITO34; sex: female; age: 61; diagnosis: non-small cell lung cancer (not otherwise specified); tumor location: lung (primary); stage: II.<br><br>Patient: ITO66; sex: female; age: 51; diagnosis: mismatch repair-deficient colorectal cancer; tumor location: lymph node metastasis (neck); stage: IV; MMR deficits (IHC): MLH1/PMS2.
Recruitment	All patients were treated at the Netherlands Cancer Institute, and tumor material and PBMCs were collected for biobanking with written informed consent. For pragmatic reasons, patients were selected for neoantigen screens based on the prior availability of in vitro-expanded TIL cultures or, for patient ITO66, organoid-induced PBMC cultures.
Ethics oversight	The study protocol (study nr. NL48824.031.14) was approved by the Medical Ethical Committee of the Netherlands Cancer Institute.

Note that full information on the approval of the study protocol must also be provided in the manuscript.

Flow Cytometry

Plots

Confirm that:

☒ The axis labels state the marker and fluorochrome used (e.g. CD4-FITC).

☒ The axis scales are clearly visible. Include numbers along axes only for bottom left plot of group (a 'group' is an analysis of identical markers).

☒ All plots are contour plots with outliers or pseudocolor plots.

☒ A numerical value for number of cells or percentage (with statistics) is provided.

Methodology

Sample preparation	Before sorting IL2-expanded patient TIL cultures into CD4+ and CD8+ T cells, cryopreserved TIL samples were thawed in RPMI 1640 medium supplemented with 10% human serum and benzonase. Samples were stained with fluorescent antibodies in MACS buffer (PBS supplemented with 0.5% bovine serum albumin and 2mM EDTA), washed with MACS buffer, and resuspended in MACS buffer for cell sorting. The same process was used to sort B cells from cryopreserved patient PBMCs for immortalization.<br><br>T cell cultures (including cultures patient TIL, TCR-transduced T cells, and T cell activation assays) were stained with fluorescent antibodies in FACS buffer (PBS supplemented with 1% bovine serum albumin and 0.05% sodium azide), washed with FACS buffer, and resuspended in FACS buffer before measurement.<br><br>Measuring IFNγ levels in culture supernatants was performed using the BD Cytometric Bead Assay following the manufacturer's instructions. Culture supernatants were incubated with capture antibody-coated beads and fluorochrome-conjugated detection antibodies, washed with BD Wash Buffer, and resuspended in BD Wash Buffer before measurement.
Instrument	BD Fusion, BD Fortessa
Software	BD FACSDiva 8.0.2 and Flowjo 10.0.7 were used for the acquisition and analysis of the other flow cytometry experiments.
Cell population abundance	CD3+CD4+ and CD3+CD8+ T cells were sorted from IL2-expanded TIL cultures, and purity of sorted populations was assessed post-sort by measuring a small alliquot of the sorted fractions.<br>Likewise, the purity of sorted CD3-CD14-CD16-CD19+ B cells from patient PBMCs was assessed by measuring a small alliquot of sorted cells post-sort.
Gating strategy	For measuring coculture experiments using TCR-transduced T cells, cell populations were first gated based on FSC-A/SSC-A, followed by gating on single cells based on SSC-A/SSC-H, gating on live cells based on IRDye, and finally on CD8 +mouseTCRbeta+ cells (in case of CD8+ T cells) or CD4+mouseTCRbeta+ cells (in case of CD4+ T cells).<br>For measuring coculture experiments using patient T cells, cell populations were gated similarly as above, but with the final gate on CD3+CD8+ or CD3+CD4+ cells.

☒ Tick this box to confirm that a figure exemplifying the gating strategy is provided in the Supplementary Information.
